# Interpretable machine learning-based predictive model for fall risk in older adults receiving maintenance hemodialysis

**DOI:** 10.3389/fmed.2026.1802938

**Published:** 2026-05-21

**Authors:** Mingjie Jiang, Meimei Zhang

**Affiliations:** 1 Department of Nursing, The First Hospital of China Medical University, Shenyang, Liaoning, China; 2 Department of Nephrology, The First Hospital of China Medical University, Shenyang, Liaoning, China

**Keywords:** fall risk, machine learning, maintenance hemodialysis, older adults, predictive model, web calculator

## Abstract

**Introduction:**

Falls are common and disabling in older adults receiving maintenance hemodialysis (MHD), yet conventional risk assessment tools often show limited predictive accuracy in this population. This study aimed to develop and validate an interpretable machine learning (ML) model to predict fall risk in older patients undergoing MHD.

**Methods:**

In this prospective study, 1,248 older adults receiving MHD were followed for 6 months. Participants were randomly divided into training and testing sets. To optimize feature selection and reduce multicollinearity, a dual-algorithm strategy combining the Least Absolute Shrinkage and Selection Operator (LASSO) and Boruta algorithms was employed. Nine ML algorithms were constructed and compared. Model performance was assessed using the area under the receiver operating characteristic curve (AUC), calibration plots, and decision curve analysis (DCA). Model interpretability was evaluated using Shapley Additive exPlanations (SHAP), and the final model was implemented as a Shiny-based web application.

**Results:**

During follow-up, 487 participants (39.0%) experienced at least one fall. Among the nine algorithms, the Categorical Boosting (CAT) model showed the best overall performance, with an AUC of 0.865 (95% confidence interval [CI]: 0.828–0.902), the highest accuracy and F1-score, and good calibration (Brier score = 0.145). DCA demonstrated that the CAT model yielded the greatest net clinical benefit across a range of threshold probabilities. SHAP analysis identified frailty, use of walking aids, and older age as the strongest contributors to fall risk.

**Conclusion:**

We developed and validated a robust and interpretable CAT-based model for predicting falls in older adults receiving MHD. By highlighting major clinical and functional risk factors and providing an accessible web-based calculator, this model may support early risk stratification and individualized fall prevention strategies in clinical practice.

## Introduction

Chronic kidney disease (CKD) has emerged as a significant global public health challenge. Characterized by a prolonged clinical course and poor prognosis, CKD severely compromises human health and quality of life (1). Epidemiological data indicate that CKD affects approximately 10% of the global population, corresponding to more than 674 million individuals worldwide (2). In China, an estimated 82 million adults are affected by CKD, representing the largest national burden globally (3). As renal function progressively declines, a substantial proportion of patients with CKD eventually advance to end-stage renal disease (ESRD), requiring renal replacement therapy (4). Among available treatment modalities, maintenance hemodialysis (MHD) remains the most widely utilized, accounting for approximately 90% of renal replacement therapy cases (5). At the same time, driven by population aging and advances in dialysis technology, the demographic profile of the MHD population is changing. Adults aged 60 and older now constitute nearly 70% of patients receiving MHD and represent the fastest-growing segment of this cohort (6).

Falls are a leading cause of unintentional injury and mortality among older adults worldwide. According to the World Health Organization (WHO), a fall is defined as an event that results in a person inadvertently coming to rest on the ground, floor, or lower level, excluding intentional changes in body position (7). Although the annual incidence of falls in the general community-dwelling geriatric population is estimated at 26.5% globally (8), older adults undergoing MHD are at substantially higher risk (9). Evidence suggests that they are more likely to experience falls and sustain fall-related injuries than their healthy counterparts (10). This heightened vulnerability is multifactorial and may reflect sarcopenia, multimorbidity, and dialysis-related physiological disturbances such as post-dialysis fatigue and hemodynamic instability (11, 12). The consequences of falls in this population can be severe, including fractures, traumatic brain injuries, and increased mortality. Beyond physical injury, falls may also trigger fear, anxiety, and depression, further reducing mobility and quality of life, thereby imposing a substantial economic burden on healthcare systems (13, 14).

Despite the urgent clinical need for fall prevention, effective risk stratification remains a persistent challenge. Current clinical practice largely relies on general fall risk assessment tools, such as the Morse Fall Scale, Hendrich II Fall Risk Model, or the STRATIFY tool (15, 16). However, these instruments are generally based on static factors or a limited number of variables and were originally developed and validated in Western populations (17). Consequently, their predictive performance in older Chinese adults undergoing MHD has been insufficiently investigated. Moreover, existing predictive models specifically designed for MHD populations have mainly used conventional logistic regression approaches (18, 19). Although informative, these traditional statistical methods assume linear relationships and may be inadequate for capturing the complex, non-linear interactions present in the high-dimensional clinical data of dialysis patients (20). Therefore, there is an urgent need to develop and validate a novel model capable of directly predicting fall probability in patients receiving MHD.

In recent years, artificial intelligence (AI) has demonstrated superior predictive performance compared with traditional statistical approaches in medical risk assessment (21). In particular, machine learning (ML) algorithms are well suited to processing high-dimensional and nonlinear data, enabling the identification of subtle pathological patterns that may otherwise remain undetected (22). This advantage is especially relevant in older populations, whose clinical profiles are often highly complex and in whom falls result from multifactorial causes that conventional statistical methods may not adequately capture (23, 24). Nevertheless, despite their predictive strength, ML-based fall prediction models are frequently limited by poor interpretability, which poses a major barrier to their implementation in clinical settings. As model complexity increases, understanding how predictions are generated becomes increasingly important for addressing the “black box” problem and strengthening clinicians’ trust in AI-assisted decision-making (25).

To date, there remains a paucity of research integrating high-performance ML algorithms with interpretability analysis specifically tailored to older Chinese adults on MHD. Therefore, the primary objective of this study was to develop and comprehensively evaluate predictive models for short-term fall-related risk using nine distinct ML algorithms. In addition, we aimed to interpret the optimal model using Shapley Additive exPlanations (SHAP) to enhance clinical transparency and identify the key drivers of risk. By translating the best-performing model into an accessible Shiny-based web calculator, this study also sought to provide clinicians with a practical and user-friendly decision-support tool for early risk stratification and targeted interventions.

## Methods

### Study design and participants

**Figure 1:**
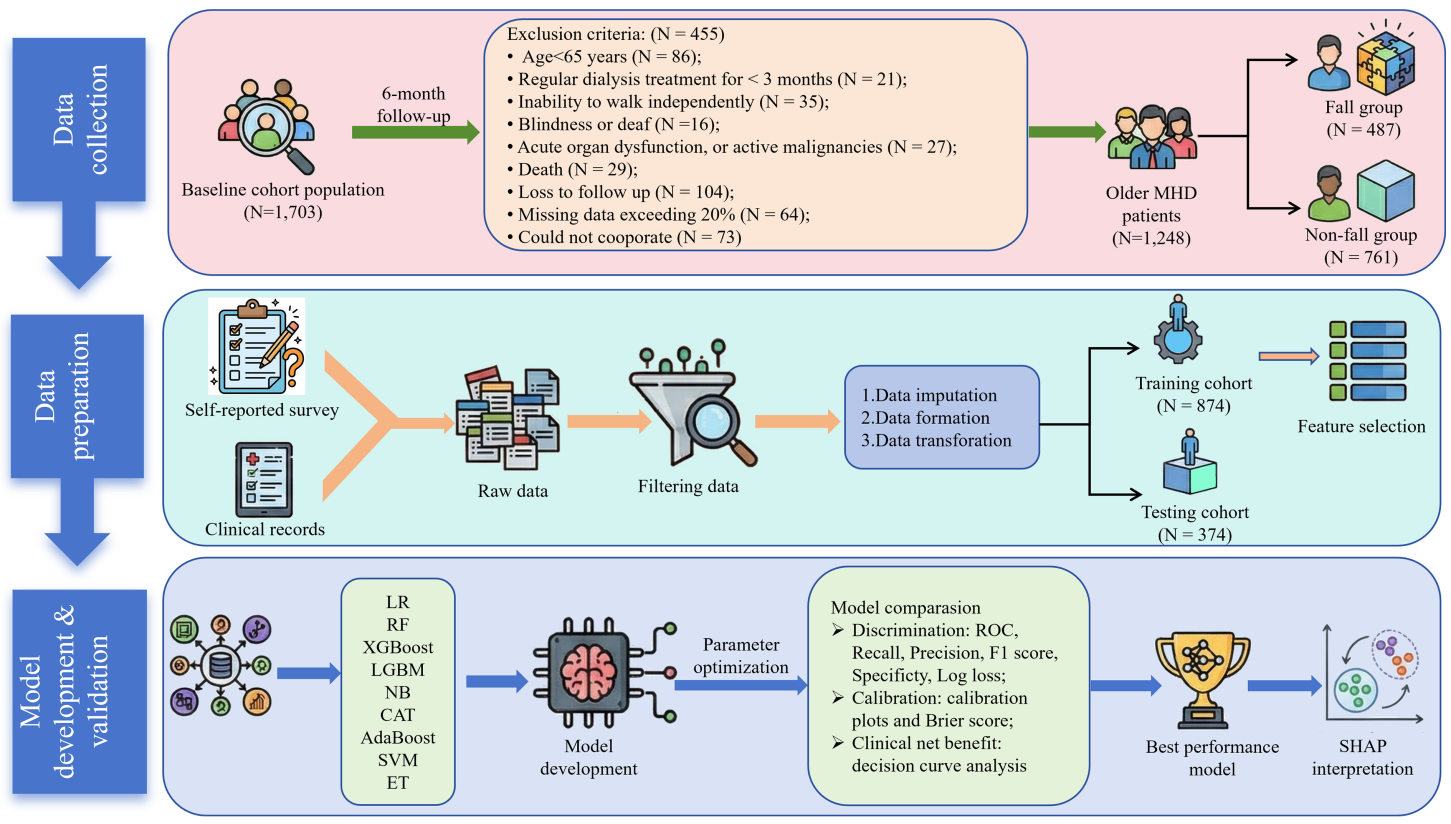
Study design and workflow.

This prospective study employed convenience sampling to recruit older patients undergoing MHD at the Hemodialysis Center of the First Affiliated Hospital of China Medical University between May 2022 and February 2025. The inclusion criteria were as follows: (1) aged ≥65 years; (2) a diagnosis of ESRD with regular dialysis treatment for ≥3 months; (3) the ability to walk independently (with or without assistive devices); and (4) provision of written informed consent for voluntary participation. Exclusion criteria included: (1) severe visual or auditory impairments preventing effective communication or assessment; (2) severe neurological disorders precluding cooperation with the survey; (3) presence of active malignancies, severe acute infection, or acute organ dysfunction; (4) loss to follow-up or death during the study period; or (5) a missing data rate exceeding 20%. This study adhered to the Declaration of Helsinki and was approved by the Ethics Committee of the First Affiliated Hospital of China Medical University (No. EC-2022-894-1). All patients provided informed consent prior to data collection. The study flow chart is shown in Figure 1.

### Measurements

#### Baseline characteristics

A structured questionnaire covering baseline information was administered to each participant upon enrollment. Data were collected through face-to-face interviews conducted by trained research nurses. The baseline characteristics were categorized into four domains: sociodemographic factors, clinical and dialysis characteristics, functional and psychosocial status, and laboratory parameters. Demographic data included age, gender, marital status, education level, monthly income, caregiver status, smoking and alcohol consumption habits, and body mass index (BMI). Dialysis-related data encompassed dialysis duration, dialysis frequency, vascular access type, intradialytic hypotension, comorbidities, and medication data. Physical and functional status assessments included the use of walking aids, any history of falls within the past year, and sensory impairments (visual and hearing), frailty, Activities of Daily Living (ADL) scores, depression, anxiety, cognitive impairment, and sleep quality. Laboratory parameters were collected prior to the mid-week hemodialysis session, including hemoglobin (Hb), albumin (Alb), serum creatinine (Scr), blood urea nitrogen (BUN), uric acid (UA), C-reactive protein (CRP), total cholesterol (TC), triglycerides (TG), and serum levels of potassium, sodium, calcium, phosphate, and intact parathyroid hormone (iPTH).

#### Assessment of cognitive function

Patients’ cognitive status was evaluated utilizing the Mini-Cog instrument. This two-part test consists of a memory registration task followed by a visuospatial distractor task (clock drawing). Specifically, patients are asked to repeat three words, draw a clock face, and then recall the words from memory. Scoring starts at a maximum of 5, with deductions applied for any unverified words (1 point per error) or an unsuccessful clock drawing (2 points). A clock drawing is deemed abnormal based on three criteria: refusal to participate, a completion time exceeding 3 min, or graphical errors. A score of < 3 was considered indicative of cognitive impairment (26).

#### Assessment of frailty

Frailty status was evaluated using the FRAIL scale, a validated and concise screening tool designed to identify frailty in clinical settings (27). The scale assesses five distinct components: fatigue, resistance, ambulation, illness burden, and weight loss. The total score ranges from 0 to 5, with higher scores reflecting greater frailty severity. Participants were categorized as frail if they obtained a score of ≥3.

#### Assessment of sleep quality

Sleep quality was assessed using the Pittsburgh Sleep Quality Index (PSQI), a validated self-report instrument used to measure sleep quality and disturbances over a 1-month period (28). The PSQI consists of 19 individual items that generate seven component scores (e.g., subjective sleep quality, sleep latency, or sleep duration). Each component is scored on a scale of 0 to 3, with total scores ranging from 0 to 21. Higher total scores reflect poorer sleep quality, and a score >5 indicates poor sleep quality. In this study, the Cronbach’s alpha was 0.924, indicating good internal consistency.

#### Measurement of depression

Depressive symptoms were evaluated using the Patient Health Questionnaire-9 (PHQ-9) (29). This instrument assesses the frequency of symptoms over the past 2 weeks across nine distinct domains (e.g., feeling down, depressed, or hopeless, and experiencing self-critical thoughts). Each item is scored on a 4-point Likert scale ranging from 0 (not at all) to 3 (nearly every day), resulting in a total score of 0 to 27. Scores of ≥10 indicate clinically significant depressive symptoms (30). In this study, the Cronbach’s alpha was 0.951, indicating good internal consistency.

#### Measurement of anxiety

Anxiety symptoms were evaluated using the 7-item Generalized Anxiety Disorder scale (GAD-7) (31). This self-reported instrument measures the frequency of symptoms over the past 2 weeks across seven domains, such as persistent nervous feelings, an inability to stop worrying, and irritability. Each item is scored on a 4-point Likert scale ranging from 0 (not at all) to 3 (nearly every day), with total scores ranging from 0 to 21. Scores of ≥10 indicate clinically significant anxiety. In this study, the Cronbach’s alpha was 0.939, indicating good internal consistency.

#### Assessment of ADL

The functional independence of participants was evaluated using the Modified Barthel Index (MBI) (32). The MBI evaluates ten specific domains of daily living (e.g., feeding, bathing, grooming, or dressing). The total score ranges from 0 to 100 (measured in 5-point increments), with higher values reflecting a greater degree of functional independence. Scores of ≤60 indicate functional dependency. In this study, the Cronbach’s alpha was 0.942, indicating good internal consistency.

### Definition of fall events and follow-up

In this study, a fall was defined as an unexpected event in which the participant inadvertently comes to rest on the ground, floor, or a lower level, not resulting from a purposeful change in position (33). In addition, fall events also encompassed instances in which participants regained stability or received assistance to avert ground contact (e.g., near-falls) (34, 35). Falls caused by overwhelming external force (e.g., violence), sudden loss of consciousness (e.g., epilepsy or syncope), or acute clinical events (e.g., stroke) were excluded.

Participants were prospectively monitored for 6 months after baseline assessment. To minimize recall bias, follow-up interviews were conducted every 2 months, either in person during dialysis sessions or by telephone. For the primary analysis, the study outcome was defined as a binary composite endpoint indicating whether a participant experienced at least one fall or near-fall event during the 6-month follow-up period. Accordingly, the prediction models were developed to estimate the 6-month risk of any fall-related event, rather than the exact timing of the first fall or the number of recurrent falls.

### Data preprocessing

To address missing values within the retained variables while strictly preventing data leakage, a sequential imputation pipeline was employed. First, the dataset was randomly partitioned into a training set (70%) and an independent testing set (30%). Subsequently, Multivariate Imputation by Chained Equations (MICE) was utilized (36). Crucially, the MICE algorithm was fitted exclusively to the training set. The distributional parameters learned from the training set were then applied to impute missing values in the training set and independently transform the hold-out testing set. This strict separation ensured that no distribution information from the testing cohort leaked into the model training phase, thereby preserving the integrity of the performance metrics.

Finally, feature transformation was performed to standardize the input space. Continuous variables were normalized using min-max scaling, which linearly transformed values into the range of [0, 1] to mitigate bias caused by varying feature magnitudes. Categorical variables were processed using one-hot encoding, transforming them into binary vectors suitable for ML algorithms.

### Feature selection

To minimize multicollinearity and enhance model interpretability, feature selection was performed using a dual-algorithm approach combining the Least Absolute Shrinkage and Selection Operator (LASSO) regression (37) and Boruta (38). Feature selection was conducted exclusively within the training set to avoid information leakage. LASSO employs an L1 regularization penalty that shrinks the coefficients of less informative variables to zero, thereby identifying a parsimonious subset of predictors. The optimal regularization parameter was determined using 10-fold cross-validation. In parallel, the Boruta algorithm was used to identify features with statistically significant predictive value. Boruta is a wrapper method based on the Random Forest classifier that compares the importance of the original variables with that of randomized “shadow features,” thereby distinguishing informative predictors from noise while preserving the underlying data distribution. The final feature set was defined as the intersection of variables selected by both LASSO and Boruta. This consensus-based strategy was used to construct the final predictive models for fall-related events in older adults receiving MHD.

### Model construction and performance assessment

In this study, nine ML algorithms were implemented to develop predictive models for fall-related risk: Logistic Regression (LR), Light Gradient Boosting Machine (LGBM), Naïve Bayes (NB), Categorical Boosting (CAT), Adaptive Boosting (AdaBoost), Extra Trees (ET), Random Forest (RF), Support Vector Machine (SVM), and Extreme Gradient Boosting (XGBoost).

To optimize model architecture and reduce the risk of overfitting, hyperparameter tuning was performed using 5-fold cross-validation within the training set. The specific parameter settings for each algorithm are provided in Supplementary Table 1. Model discrimination was primarily assessed using the area under the receiver operating characteristic curve (AUC), reported with 95% confidence intervals (CI). Additional performance metrics included accuracy, recall (sensitivity), precision, specificity, F1-score, and Log-Loss. Calibration was evaluated using calibration curves and the Brier score, which measures the mean squared difference between predicted probabilities and observed outcomes. Decision Curve Analysis (DCA) was also performed to assess the potential clinical utility of each model by estimating net benefit across a range of threshold probabilities.

### Model interpretation via SHAP

To address the “black-box” nature of the developed ML models and enhance clinical transparency, we employed the SHAP framework for model interpretation (39). Based on cooperative game theory, SHAP provides a mathematically rigorous method for assigning each feature a consistent and locally accurate contribution value—the SHAP value—to each individual prediction. A SHAP value represents the average marginal contribution of a given feature to the model’s output across all possible feature combinations. In this study, SHAP quantifies both the direction and magnitude of each feature’s effect on the predicted outcome. This framework supports global interpretability by ranking feature importance across the entire study population and local interpretability by illustrating how individual clinical characteristics influence predictions for specific patients.

### Web-based risk calculator

To support clinical implementation of the developed prediction model, the best-performing algorithm was integrated into a Shiny-based web platform. The web calculator allows users to input the final model variables and automatically returns an individualized predicted probability of a fall-related event during the 6-month follow-up period in older adults receiving MHD.

### Sensitivity analysis

To assess the robustness of our primary findings and address potential outcome heterogeneity and recall bias associated with near-falls, a sensitivity analysis was conducted using actual falls only as the endpoint. Following the exact methodological pipeline as the primary analysis, we utilized the previously identified predictive variables and the optimal model architecture. The restricted dataset was re-partitioned into a 70% training subset of the complete-case (hard fall only) cohort and subsequently evaluated on the remaining 30% independent testing subset.

### Statistical analysis

All statistical analyses and data visualizations were performed using R software (version 4.4.3). Data management and preprocessing were conducted utilizing the “tidyverse” and “data.table” packages. Missing-data imputation was performed using the “mice” package. Feature selection was conducted by the “glmnet” package for LASSO and the “Boruta” package for Boruta. Hyperparameter tuning and model training were implemented using the “caret” package. The ML algorithms were fitted using the “glmnet” for LR, “catboost” for CAT, “xgboost” for XGBoost, “lightgbm” for LGBM, “e1071” for NB and SVM, “extraTrees” for ET, “randomForest” for RF, and “adabag” for AdaBoost. Model performance was evaluated using the “pROC” package for ROC, the “rms” package for calibration, and the “rmda” package for DCA. SHAP was performed using “shapviz.”

Given the non-normal distribution of the clinical parameters in this cohort, continuous variables were presented as medians with interquartile ranges (IQR) and were compared using the Mann–Whitney U test. Categorical variables were expressed as frequencies and percentages (*N*, %). Intergroup comparisons for categorical data were conducted using the Pearson chi-square test. A two-tailed *p*-value <0.05 was considered statistically significant.

## Results

### Baseline characteristics

The study flow and participant selection process are shown in Figure 1. A total of 1,248 older adults undergoing MHD were included in the final analysis. The median age of the cohort was 73 years (IQR: 69–78), and 60.7% were male. Regarding dialysis-related characteristics, 30.7% of patients had a dialysis vintage exceeding 5 years, and 74.0% received MHD more than twice weekly. Furthermore, polypharmacy (88.6%) and visual impairment (57.4%) were common in the cohort. During the follow-up period, 487 participants (39.0%) experienced a fall-related event.

As shown in Table 1, participants with fall-related events were significantly older (*p* < 0.001) and had a higher prevalence of comorbidities, including prior stroke (*p* < 0.001), intradialytic hypotension (*p* = 0.035), and orthopedic disease (*p* < 0.001). They were also more likely to have polypharmacy (*p* < 0.001), use walking aids (*p* < 0.001), and report a history of falls in the previous year (*p* = 0.023). Functional and sensory impairments, including visual impairment (*p* < 0.001), cognitive impairment (*p* < 0.001), frailty (*p* < 0.001), and functional dependency (*p* < 0.001) were also significantly more frequent in the fall group. In addition, the fall group had lower hemoglobin levels (*p* = 0.022), and higher iPTH levels (*p* = 0.016) (Table 1).

**Table 1:** Comparison of baseline characteristics between participants with or without falls.

Characteristics	Total (*N* = 1,248)	Fall group (*N* = 487)	Non-fall group (*N* = 761)	Statistics	*p*-value
Age	73.00 [69.00, 78.00]	76.00 [71.00, 80.00]	72.00 [68.00, 76.00]	−9.019	<0.001
Gender					
Male	758 (60.7%)	298 (61.2%)	460 (60.4%)	0.069	0.793
Female	490 (39.3%)	189 (38.8%)	301 (39.6%)		
BMI (kg/m^2^)	23.30 [20.00, 27.90]	23.20 [20.00, 28.30]	23.30 [20.10, 27.70]	−0.134	0.893
Education level					
Below high school	910 (72.9%)	357 (73.3%)	553 (72.7%)	0.061	0.804
High school or above	338 (27.1%)	130 (26.7%)	208 (27.3%)		
Marital status					
Married	835 (66.9%)	328 (67.4%)	507 (66.6%)	0.071	0.790
Single/divorced/widowed	413 (33.1%)	159 (32.6%)	254 (33.4%)		
Caregivers					
Family member	1,129 (90.5%)	438 (89.9%)	691 (90.8%)	0.257	0.613
Nursing assistant or others	119 (9.5%)	49 (10.1%)	70 (9.2%)		
Monthly income					
<3,000 RMB	552 (44.2%)	210 (43.1%)	342 (44.9%)	0.399	0.819
3,000–5,000 RMB	568 (45.5%)	226 (46.4%)	342 (44.9%)		
>5,000 RMB	128 (10.3%)	51 (10.5%)	77 (10.1%)		
Current smoking	116 (9.3%)	45 (9.2%)	71 (9.3%)	0.003	0.958
Current alcohol consumption	95 (7.6%)	38 (7.8%)	57 (7.5%)	0.041	0.839
Dialysis duration					
<2 years	272 (21.8%)	106 (21.8%)	166 (21.8%)	0.549	0.760
2–5 years	593 (47.5%)	237 (48.7%)	356 (46.8%)		
>5 years	383 (30.7%)	144 (29.6%)	239 (31.4%)		
Dialysis frequency per week					
≤2 times	325 (26.0%)	123 (25.3%)	202 (26.5%)	0.256	0.613
>2 times	923 (74.0%)	364 (74.7%)	559 (73.5%)		
Vascular access type					
Arteriovenous fistula	901 (72.2%)	355 (72.9%)	546 (71.7%)	0.195	0.659
Central venous catheter	347 (27.8%)	132 (27.1%)	215 (28.3%)		
Intradialytic hypotension	301 (24.1%)	133 (27.3%)	168 (22.1%)	4.445	0.035
Diabetes	478 (38.3%)	182 (37.4%)	296 (38.9%)	0.292	0.589
Hypertension	986 (79.0%)	385 (79.1%)	601 (79.0%)	0.001	0.973
Stroke	215 (17.2%)	123 (25.3%)	92 (12.1%)	36.106	<0.001
Cardiovascular disease	325 (26.0%)	122 (25.1%)	203 (26.7%)	0.407	0.524
Orthopedic disease	551 (44.2%)	295 (60.6%)	256 (33.6%)	87.373	<0.001
Sedative-hypnotic medications	439 (35.2%)	167 (34.3%)	272 (35.7%)	0.274	0.601
Polypharmacy	1,106 (88.6%)	457 (93.8%)	649 (85.3%)	21.566	<0.001
Using of walking aids	461 (36.9%)	256 (52.6%)	205 (26.9%)	83.733	<0.001
History of falls in the past year	352 (28.2%)	155 (31.8%)	197 (25.9%)	5.175	0.023
Visual impairment	716 (57.4%)	348 (71.5%)	368 (48.4%)	64.796	<0.001
Hearing impairment	411 (32.9%)	165 (33.9%)	246 (32.3%)	0.325	0.569
Cognitive impairment	353 (28.3%)	201 (41.3%)	152 (20.0%)	66.415	<0.001
Poor sleep quality	947 (75.9%)	378 (77.6%)	569 (74.8%)	1.316	0.251
Frailty	521 (41.7%)	282 (57.9%)	239 (31.4%)	85.749	<0.001
Depression	778 (62.3%)	318 (65.3%)	460 (60.4%)	2.977	0.084
Anxiety	721 (57.8%)	276 (56.7%)	445 (58.5%)	0.395	0.530
ADL dependency	835 (66.9%)	384 (78.9%)	451 (59.3%)	51.450	<0.001
Laboratory parameters					
Hemoglobin (g/L)	114.50 [100.00, 132.00]	111.00 [99.00, 129.00]	117.00 [100.00, 133.00]	−2.284	0.022
Albumin (g/L)	38.00 [31.00, 44.00]	38.00 [31.00, 44.00]	38.00 [32.00, 44.00]	−0.210	0.834
Serum potassium (mmol/L)	4.70 [4.20, 5.20]	4.80 [4.20, 5.20]	4.70 [4.20, 5.20]	−0.647	0.518
Serum phosphate (mmol/L)	1.61 [1.38, 1.98]	1.61 [1.36, 1.96]	1.61 [1.39, 2.00]	−0.745	0.457
Serum calcium (mmol/L)	1.78 [1.57, 2.00]	1.78 [1.58, 2.01]	1.78 [1.57, 2.00]	−0.124	0.901
Serum sodium (mmol/L)	142.00 [138.00, 146.00]	141.00 [138.00, 146.00]	142.00 [138.00, 146.00]	−0.182	0.856
iPTH (pg/mL)	249.00 [162.00, 377.75]	255.00 [174.00, 394.00]	241.00 [155.00, 374.00]	−2.410	0.016
CRP (mg/L)	8.52 [6.17, 10.52]	8.65 [6.20, 10.54]	8.49 [6.11, 10.50]	−0.724	0.469
TC (mmol/L)	4.10 [3.50, 4.80]	4.10 [3.60, 4.80]	4.10 [3.50, 4.80]	−0.212	0.832
TG (mmol/L)	1.80 [1.60, 2.10]	1.80 [1.70, 2.10]	1.80 [1.60, 2.10]	−0.260	0.795
BUN (mmol/L)	21.70 [17.70, 27.10]	21.90 [17.50, 27.00]	21.60 [17.85, 27.15]	−0.710	0.478
UA (mg/dL)	9.60 [8.10, 11.10]	9.60 [8.00, 11.20]	9.60 [8.10, 11.00]	−0.313	0.754
Scr (mg/dL)	7.50 [5.80, 9.20]	7.50 [5.80, 9.20]	7.40 [5.80, 9.20]	−0.220	0.826

Data are presented as median [interquartile ranges] or *N* (%). BMI, body mass index; RMB, Renminbi; ADL, activities of daily living; iPTH, intact parathyroid hormone; CRP, C-reactive protein; TC, total cholesterol; TG, triglyceride; BUN, blood urea nitrogen; UA, uric acid; Scr, serum creatinine.

Among the participants who died or were lost to follow-up (*n* = 133), 29 died before completion of follow-up and 104 were lost to follow-up, resulting in unavailable 6-month outcome ascertainment. Baseline comparisons between included (*n* = 1,248) and excluded participants (*n* = 133) are shown in Supplementary Table S2 and did not reveal statistically significant differences in the measured baseline variables.

### Variable selection by dual-algorithm approach

**Figure 2:**
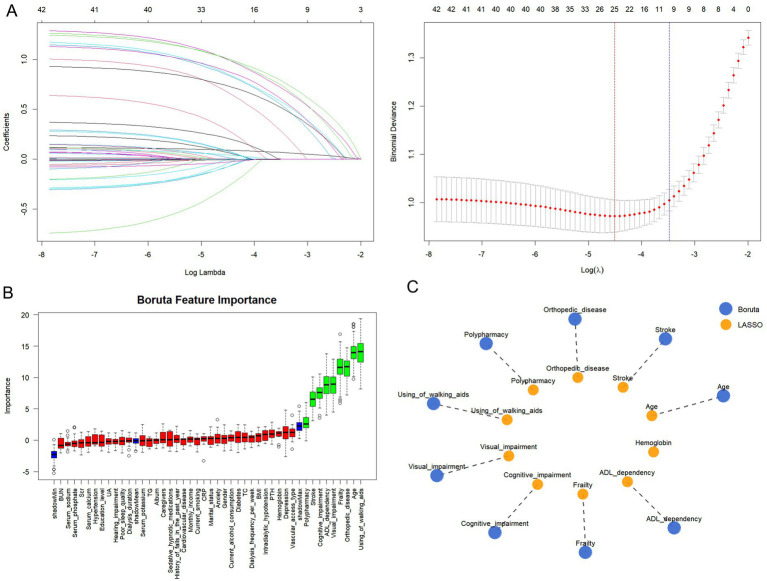
Feature selection using a dual-algorithm approach. **(A)** Selection of the optimal tuning parameter (lambda) in the LASSO regression model using 10-fold cross-validation. **(B)** Variable importance ranking generated by the Boruta algorithm. **(C)** Visualization of the consensus variables selected by both the LASSO and Boruta algorithms.

The 1,248 participants were randomly partitioned into a training set (*N* = 874) and a testing set (*N* = 374). Baseline clinical characteristics showed no statistically significant differences between the training and testing cohorts (all *p* > 0.05), as detailed in Supplementary Table S3. LASSO regression was first applied for preliminary feature selection. Using 10-fold cross-validation, lambda.1se (0.031) was selected as the optimal regularization parameter. This procedure retained 10 variables (Figure 2A): age, stroke, orthopedic disease, polypharmacy, using of walking aids, visual impairment, cognitive impairment, frailty, ADL dependency, and hemoglobin. To further assess the robustness of the selected predictors, Boruta analysis was subsequently performed. The Boruta results showed substantial overlap with variables retained by LASSO regression (Figure 2B). Ultimately, a consensus set of nine discriminative variables was identified by both methods and used for final model construction: age, stroke, orthopedic disease, polypharmacy, using of walking aids, visual impairment, cognitive impairment, frailty, and ADL dependency (Figure 2C). The pre-imputation missing counts and missingness rates for the final selected predictors are presented in Supplementary Table S4. All final predictors showed relatively low levels of missingness before imputation.

### Model performance

**Figure 3:**
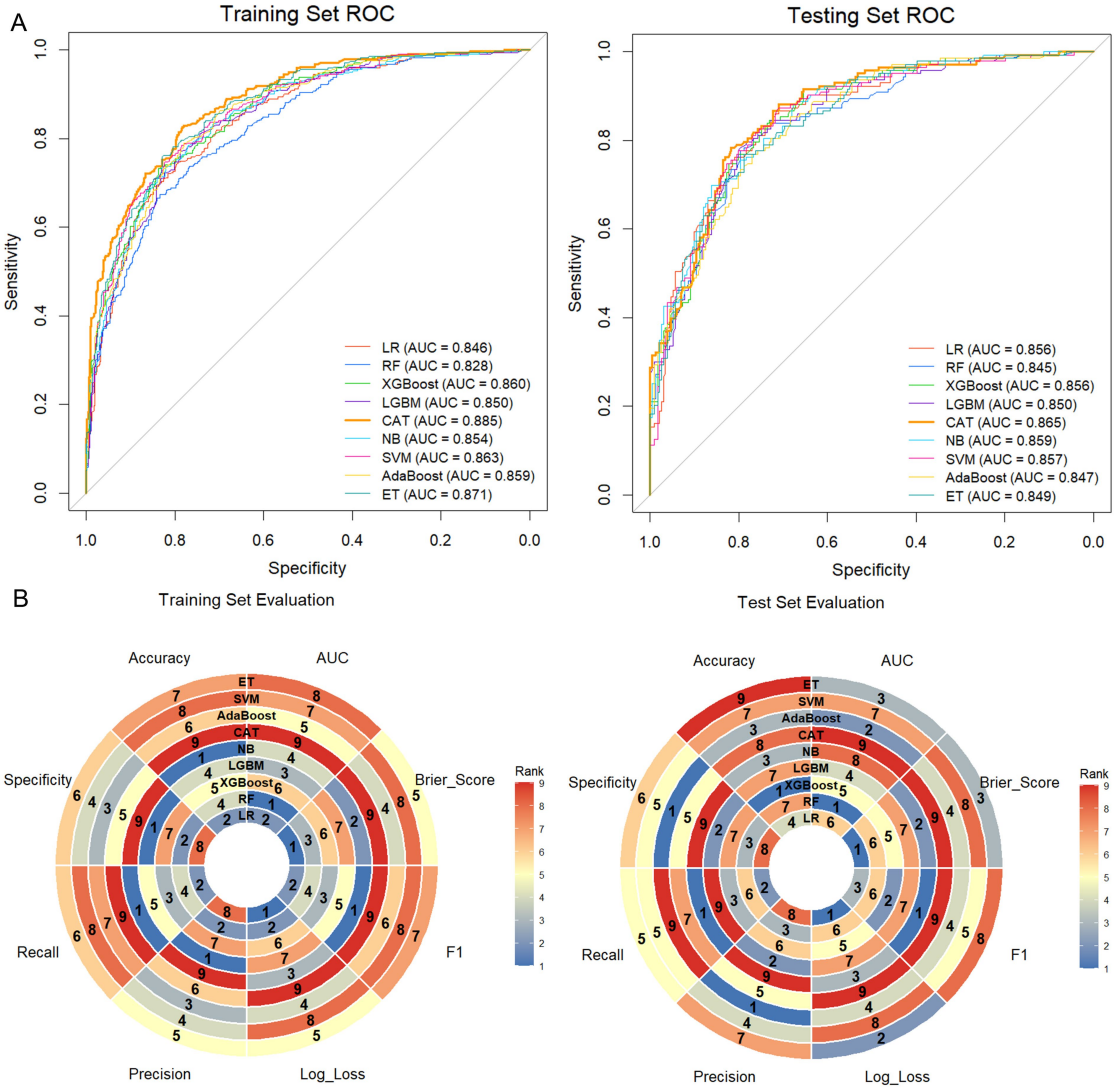
Comparative assessment of model performance. **(A)** ROC curves of the nine ML models in the training and testing sets. **(B)** Radar plots summarizing the overall performance metrics of each model in the training and testing sets.

**Figure 4:**
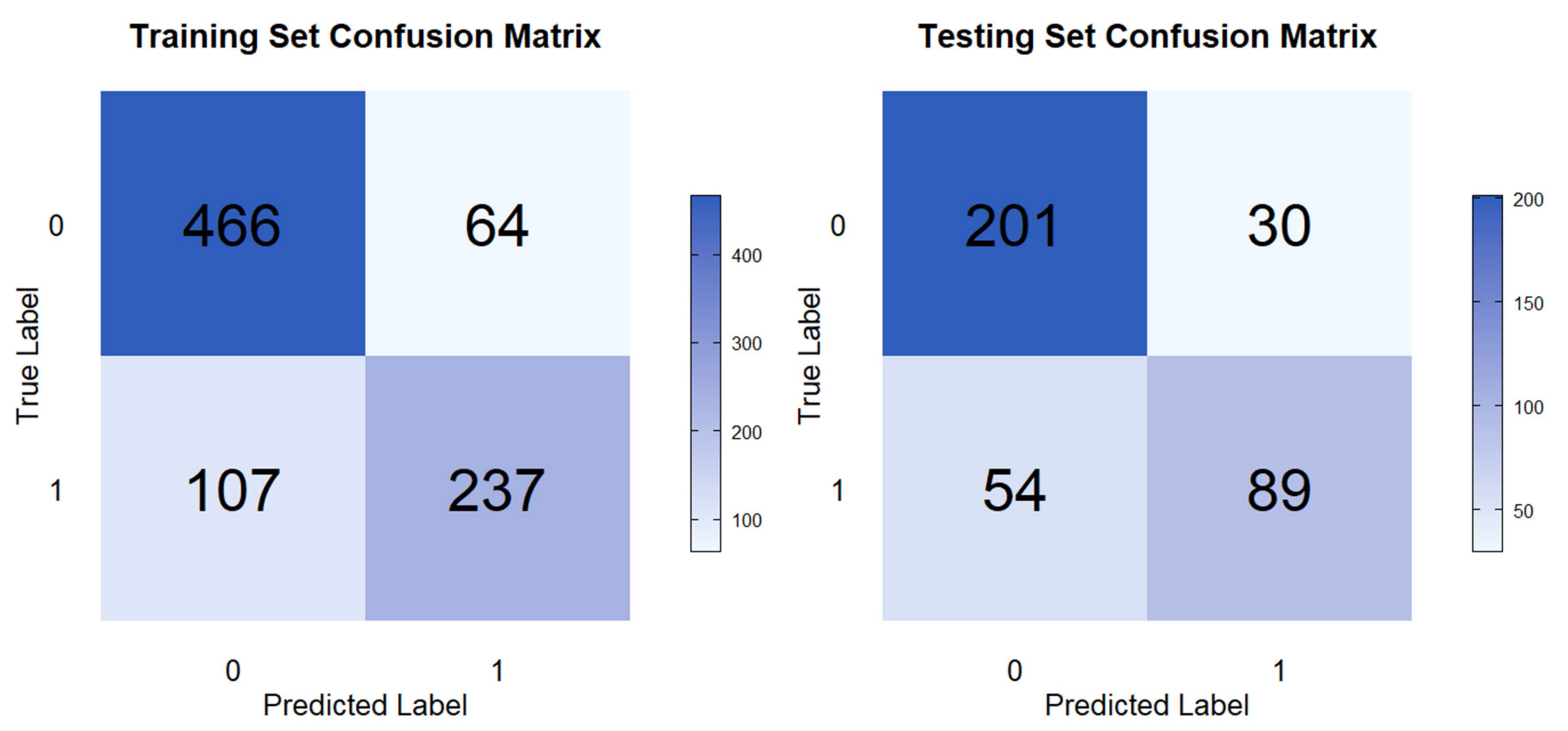
Confusion matrices of the CAT model. Visualization of the classification performance of the CAT model in the training and testing sets.

The performance metrics for all ML models are summarized in Table 2 and illustrated in Figure 3. Among the nine algorithms, the CAT model showed the best overall discriminative performance, achieving the highest AUC in both the training set (AUC: 0.885; 95% CI: 0.863–0.907) and the testing set (AUC: 0.865; 95% CI: 0.828–0.902) (Figure 3A). Furthermore, comprehensive performance assessment indicated that the CAT model outperformed other algorithms across multiple metrics. In the training set, it achieved the highest accuracy (0.804), recall (0.689), and F1-score (0.735), alongside the lowest Log-Loss (0.423) (Figure 3B). In the testing set, CAT retained the lowest Log-Loss (0.444) and the highest F1-score (0.679), supporting its stability and generalization performance (Figure 3B). Although the NB model showed slightly higher specificity and precision in the testing set, its poor recall (0.413) and relatively low F1-score (0.565) suggested limited ability to correctly identify patients who experienced fall-related events. Consistently, the confusion matrices further demonstrated that the CAT model effectively distinguished participants with and without fall-related events in both the training and testing cohorts (Figure 4).

**Table 2:** The predictive performance of the nine machine learning models.

Model	Accuracy	AUC (95% CI)	Recall	Precision	F1-score	Specificity	Log-Loss	Brier score
Training set								
LR	0.763	0.846 (0.820–0.872)	0.526	0.804	0.636	0.917	0.528	0.173
RF	0.768	0.828 (0.800–0.855)	0.642	0.734	0.685	0.849	0.504	0.164
XGBoost	0.773	0.860 (0.835–0.884)	0.578	0.790	0.668	0.900	0.481	0.156
LGBM	0.768	0.850 (0.824–0.875)	0.648	0.731	0.687	0.845	0.471	0.154
NB	0.748	0.854 (0.829–0.879)	0.445	0.841	0.582	0.945	0.503	0.167
CAT	0.804	0.885 (0.863–0.907)	0.689	0.787	0.735	0.879	0.423	0.136
AdaBoost	0.776	0.859 (0.835–0.884)	0.657	0.743	0.698	0.853	0.493	0.160
SVM	0.794	0.863 (0.839–0.888)	0.677	0.772	0.721	0.870	0.450	0.145
ET	0.792	0.871 (0.848–0.895)	0.654	0.781	0.712	0.881	0.487	0.157
Testing set								
LR	0.767	0.856 (0.817–0.895)	0.503	0.818	0.623	0.931	0.519	0.170
RF	0.770	0.845 (0.805–0.885)	0.615	0.739	0.672	0.866	0.476	0.155
XGBoost	0.751	0.856 (0.818–0.894)	0.524	0.750	0.617	0.892	0.481	0.157
LGBM	0.770	0.850 (0.811–0.889)	0.629	0.732	0.677	0.857	0.462	0.152
NB	0.757	0.859 (0.822–0.897)	0.413	0.894	0.565	0.970	0.499	0.166
CAT	0.775	0.865 (0.828–0.902)	0.622	0.748	0.679	0.870	0.444	0.145
AdaBoost	0.757	0.847 (0.808–0.886)	0.629	0.703	0.664	0.835	0.496	0.162
SVM	0.770	0.857 (0.818–0.895)	0.608	0.744	0.669	0.870	0.460	0.149
ET	0.778	0.849 (0.810–0.888)	0.608	0.763	0.677	0.883	0.499	0.163

AUC, area under the receiver operating characteristic curve; CI, confidence interval; LR, logistic regression; RF, Random Forest; XGBoost, extreme gradient boosting; LGBM, light gradient boosting machine; NB, Naive Bayes; CAT, categorical boosting; AdaBoost, adaptive boosting; SVM, support vector machine; ET, extra trees.

**Figure 5:**
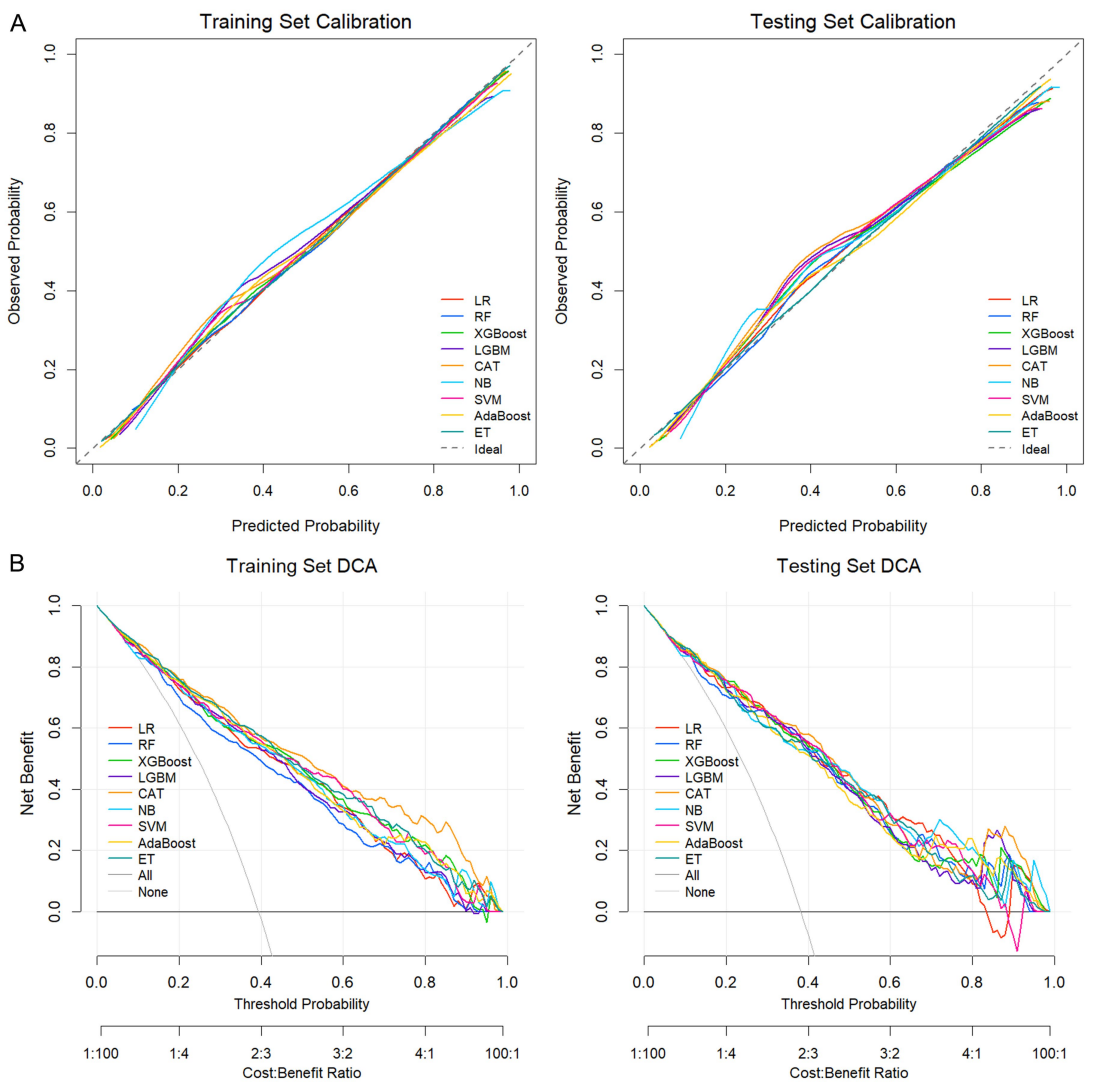
Calibration and clinical utility analyses. **(A)** Calibration curves of the nine ML models in the training and testing sets. **(B)** DCA showing the net benefit of the models across a range of threshold probabilities in the training and testing sets.

Calibration curves showed generally good agreement between predicted probabilities and observed outcomes across models (Figure 5A). Notably, the CAT model had the lowest Brier scores in both the training (0.136) and testing (0.145) sets, indicating the best probabilistic accuracy among the candidate models (Table 2). Additionally, DCA further supported the clinical utility of the CAT model, which provided the highest net benefit across the widest range of threshold probabilities in comparison with the other algorithms (Figure 5B).

### Sensitivity analysis

To examine whether inclusion of near falls influenced model performance, we performed a sensitivity analysis restricted to actual falls only. During follow-up, 341 participants experienced at least one actual fall, corresponding to an incidence of 27.3%. In the independent testing cohort, the CAT model achieved an AUC of 0.816 (95% CI: 0.770–0.862), an accuracy of 0.751, and a Brier score of 0.158 (Supplementary Table S5; Supplementary Figure S1). Although discrimination and calibration were modestly attenuated compared to the primary composite endpoint analysis, the model retained acceptable predictive performance. These findings suggest that the main conclusions were not solely dependent on the inclusion of near-fall events.

### Model interpretability analysis

**Figure 6:**
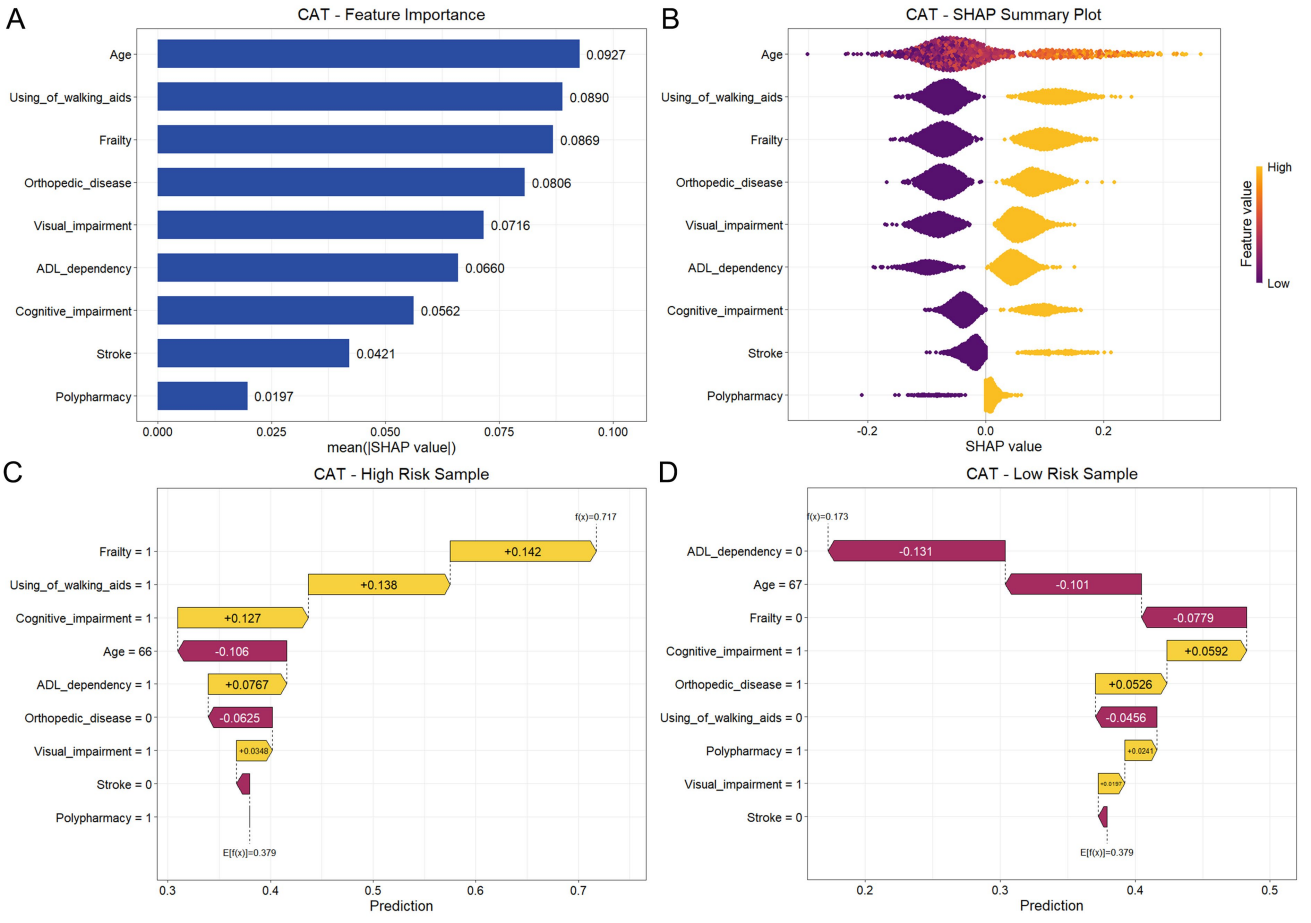
SHAP-based interpretability analysis of the CAT model. **(A)** Global feature importance ranking based on the mean absolute SHAP values, identifying the most influential predictors of fall risk. **(B)** SHAP beeswarm plot illustrating the distribution of SHAP values for each feature. Yellow dots indicate high feature values, whereas purple dots indicate low feature values; the horizontal position represents the impact on model output, with positive values indicating increased fall-related risk. **(C)** SHAP waterfall plot for a representative high-risk case, showing the contribution of each feature to a final prediction from the base value (E[f(x)]). **(D)** SHAP waterfall plot for a representative low-risk case.

To improve the transparency of the final predictive model, we employed SHAP to quantify the contribution of individual features within the CAT algorithm. Figure 6A illustrated the global feature importance ranking for discriminating fall risk in the older MHD population. The analysis identified age, using of walking aids, frailty, orthopedic disease, and visual impairment as the top five most influential predictors. Subsequently, SHAP beeswarm plots were generated to visualize the directional impact of these variables (Figure 6B). In this plot, the horizontal axis represented the SHAP value, while the color gradient indicated the feature value (yellow for high and purple for low). A larger absolute deviation from zero signified a stronger impact on the model’s output. The results demonstrated that advanced age, using of walking aids, frailty, orthopedic disease, visual impairment, ADL dependency, cognitive impairment, stroke, and polypharmacy were all associated with positive SHAP values, indicating a direct correlation with increased fall-related risk.

To further illustrate individual-level predictions, SHAP waterfall plots were generated for representative cases. In these plots, yellow bars (extending to the right) indicated features that increased fall probability, whereas red bars (extending to the left) indicated protective factors. Figure 6C displayed a representative high-risk case in which the model predicted a fall-related event probability of 0.717, significantly exceeding the baseline expectation (E[f(x)] = 0.379). The main contributors to this elevated risk were frailty (SHAP = +0.142), using of walking aids (SHAP = +0.138), cognitive impairment (SHAP = +0.127), and high ADL dependency (SHAP = +0.0767). Conversely, Figure 6D illustrated a representative low-risk case with a predicted probability of 0.173. In this patient, the predicted risk was reduced mainly by low ADL dependency (SHAP = −0.131), younger age (SHAP = −0.101), and absence of frailty (SHAP = −0.0779).

### Implementation of the web calculator

**Figure 7:**
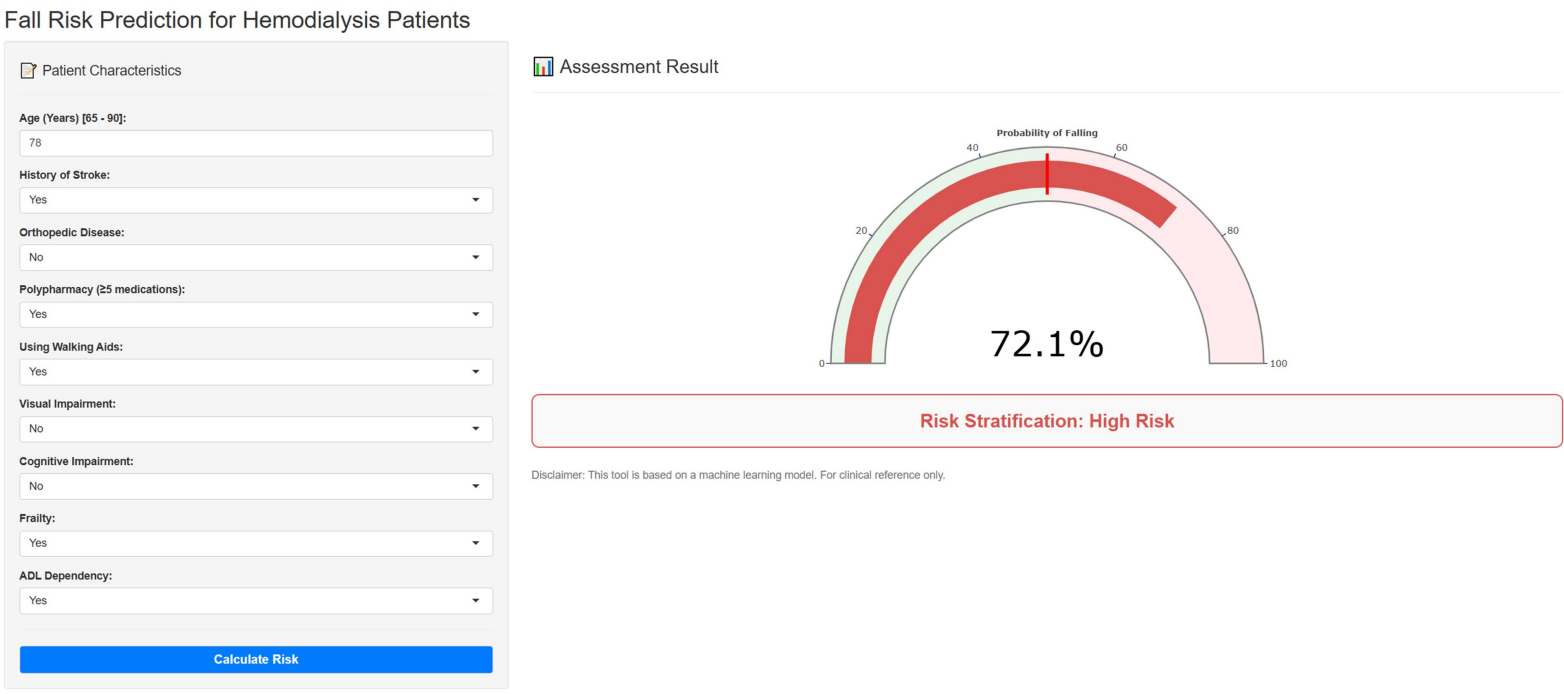
Screenshot of the Shiny-based web calculator for individualized prediction of the 6-month risk of fall-related events in older adults receiving MHD.

As illustrated in Figure 7, the final CAT model was deployed as an interactive Shiny web application to facilitate clinical use. By entering the values of the nine predictors included in the final model, clinicians can obtain an individualized predicted probability of experiencing a fall-related event within 6 months in older adults receiving MHD. This tool may help clinicians identify high-risk patients early and support targeted fall-prevention strategies in routine dialysis care. The web application is available online at the following link: https://mlwebtool.shinyapps.io/MHDfallrisk/.

## Discussion

Older adults with ESRD receiving MHD are highly vulnerable to falls, which are associated with fractures, hospitalization, functional decline, and mortality (40). In this context, early identification of patients at increased short-term risk may help prioritize preventive assessment and intervention. In the present study, we analyzed a cohort of 1,248 older adults receiving MHD and applied a dual-algorithm feature selection strategy to identify key predictors of fall-related events. By developing and comparing nine different ML models, we found that the CAT algorithm showed the best overall performance, achieving an AUC of 0.865 together with favorable calibration and decision-analytic utility. In addition, SHAP analysis improved model interpretability by quantifying the direction and magnitude of each predictor’s contribution, thereby linking model output to clinically recognizable risk factors.

Previous approaches to fall-risk stratification in MHD populations have predominantly relied on traditional LR- or nomogram-based frameworks. For example, Liu et al. reported moderate discrimination (AUC: 0.663–0.773) (18), and Li et al. developed a similar tool for older MHD patients (19). Although these approaches are clinically accessible, they may be limited when predictor-outcome relationships are nonlinear or involve higher-order interactions, as is often the case in geriatric syndromes (41). In contrast, ML methods can more flexibly accommodate complex relationships within structured clinical datasets (42). Prior studies in broader geriatric settings have also suggested the value of ML for fall prediction. For instance, Ikeda et al. applied an interpretable XGBoost model to examine longitudinal fall risk in community-dwelling older adults, and Polus et al. reported favorable performance of SVM-based prediction in a post-arthroplasty cohort (43). Our study extends this line of research to older adults receiving MHD and, to our knowledge, represents one of the first systematic comparisons of multiple interpretable ML algorithms for fall-related event prediction in this particularly high-risk population. Among the algorithms assessed, the CAT model displayed superior performance, achieving an AUC of 0.865 and an F1-score of 0.679.

The heterogeneous performance of the evaluated algorithms was broadly consistent with the characteristics of this clinical dataset. Although simpler probabilistic and linear models such as NB and LR achieved reasonably high AUCs, they performed less favorably on threshold-dependent and calibration-related metrics. In particular, NB showed relatively low recall and F1-score, indicating limited ability to identify a substantial proportion of patients who subsequently experienced fall-related events. This pattern is methodologically plausible because NB relies on a conditional independence assumption that is unlikely to hold in older hemodialysis patients, in whom frailty, age, using of walking aids, and comorbidity burden are closely interrelated (44). LR also showed relatively low sensitivity and less favorable calibration than the leading models, suggesting that a simple linear framework may not fully capture the nonlinear and interactive determinants of fall risk in this cohort (20). By contrast, tree-based boosting methods, especially CAT, appear better suited to structured clinical tabular data because they can model nonlinear relationships and higher-order interactions more flexibly, which likely contributed to their more balanced overall performance (45).

A major strength of the present study is that the final model was not only predictive but also interpretable. To improve transparency and facilitate clinical understanding, we applied the SHAP framework to quantify both the magnitude and direction of each feature’s contribution to the model output (46). SHAP analysis identified a distinct set of discriminative features associated with fall-related risk, including age, use of walking aids, frailty, orthopedic disease, visual impairment, ADL dependency, cognitive impairment, stroke, and polypharmacy. Notably, these retained predictors were predominantly geriatric and functional in nature rather than explicitly dialysis-related. This should not be interpreted as suggesting that dialysis-related factors are clinically unimportant. Rather, within the present dataset and feature selection framework, these geriatric-functional indicators appeared to contribute more strongly to short-term fall-related events than the dialysis-related variables in our cohort. This pattern is clinically plausible because falls in older adults receiving MHD arise from multidimensional vulnerability, in which frailty, mobility limitations, and sensory-cognitive deficits may serve as more proximal determinants of near-term instability (10, 47). Although dialysis-related factors may still influence fall susceptibility indirectly through mechanisms such as intradialytic hemodynamic instability, post-dialysis fatigue, and bone disorders, our findings suggest that functional and geriatric status may provide superior discriminative value for near-term risk stratification in this vulnerable population (48, 49).

Consistent with previous literature (50), advanced age emerged as a major predictor of fall-related risk. Prior cohort studies have similarly shown that fall incidence increases substantially with age, particularly among the oldest-old population. This association is likely multifactorial and may reflect the cumulative burden of chronic comorbidity, age-related physiological decline, impaired sensory integration, and increased environmental vulnerability (51). Using of walking aids was also identified as a strong predictor. Rather than acting as a direct cause of falls, reliance on assistive devices may serve as a surrogate marker for underlying gait instability, muscle weakness, or balance impairment. This interpretation is consistent with previous findings in community-dwelling adults, in whom use of assistive devices has been associated with future fall events (52, 53). From a practical perspective, the need for a walking aid may serve as a readily identifiable clinical warning sign in the dialysis setting.

Frailty emerged as one of the most important predictors of fall-related risk in the present model. Frailty is a multidimensional syndrome characterized by reduced physiological reserves and diminished resilience to stressors, and it has repeatedly been associated with adverse outcomes, including falls, in older adults (54). Our findings are consistent with previous reports identifying frailty as a key risk marker in dialysis populations (55). In patients with ESRD, protein-energy wasting, chronic inflammation, sarcopenia, and treatment burden may all contribute to physical frailty and reduced functional capacity. These mechanisms may help explain why frailty was strongly associated with fall-related risk in this cohort. Moreover, orthopedic disease also contributed meaningfully to fall-related risk. Conditions such as osteoporosis, osteoarthritis, and prior fragility fractures may compromise musculoskeletal stability and increase vulnerability to both falls and fall-related injury. In patients receiving long-term hemodialysis, the skeletal system may be further affected by chronic kidney disease-mineral and bone disorder and dialysis-related bone fragility (53, 56). Accordingly, close monitoring of bone health may be important in reducing structural vulnerability in this population.

Visual impairment emerged as another critical predictor of fall-related risk. Visual deficits, which are common among patients receiving hemodialysis (57), may compromise postural stability, environmental scanning, and obstacle avoidance—functions that are often already impaired in this population because of concurrent vestibular, neurological, and musculoskeletal dysfunctions (58). In addition, ADL dependency likely reflects a broader decline in physical function and self-care capacity, which has consistently been associated with falls in geriatric populations (59). The physiological aging process is intrinsically linked to progressive decline in physical capacity, resulting in functional insufficiency and impaired self-care abilities. This trajectory may be further accelerated in older adults undergoing chronic MHD, with important consequences for functional independence and quality of life. Together, these findings suggest that physical vulnerability and impaired functional independence are central components of fall-related risk in older MHD patients.

Cognitive impairment, prior stroke, and polypharmacy also emerged as relevant predictors. Cognitive deficits inherently compromise executive function, attention, processing speed, and hazard recognition, thereby escalating susceptibility to gait disturbance and mechanical falls (60). This vulnerability may be particularly relevant in the hemodialysis population, where recurrent intradialytic hemodynamic fluctuations, cerebrovascular disease, and cumulative treatment burden synergistically accelerate neurocognitive decline (61). A history of stroke further amplifies this risk through persistent motor deficits and executive dysfunction (62). In addition, although polypharmacy is often unavoidable in older adults with ESRD, it may increase fall susceptibility through adverse drug events, drug–drug interactions, and the broader multimorbidity burden it reflects (63). Collectively, these findings suggest that fall-related events in older MHD patients arise from the interaction of multiple functional, neurological, and treatment-related vulnerabilities rather than from a single isolated factor.

In this study, near-falls were included in the primary composite endpoint because they represent clinically meaningful manifestations of postural instability and balance impairment, even in the absence of ground contact or injury (34). Among older adults receiving MHD, near-falls likely reflect the same underlying vulnerabilities that predispose to actual falls, including frailty, sarcopenia, orthostatic symptoms, gait dysfunction, and dialysis-related hemodynamic fluctuations (64). Importantly, near-falls may precede overt falls and can therefore serve as an early marker of progressive functional decline and increasing fall susceptibility (35). By incorporating near-falls into the primary analysis, we aimed to capture instability-related events more comprehensively and improve the sensitivity of risk identification. This approach aligns with the clinical goal of early detection and prevention in a population that is particularly vulnerable to mobility-related adverse outcomes. At the same time, we acknowledge that near-falls and actual falls are related but distinct outcomes. Their combination may introduce some degree of outcome heterogeneity and may be more susceptible to self-report and recall bias. To address these concerns, events were prospectively assessed at 2-month intervals to reduce recall error, and we additionally performed a sensitivity analysis restricted to actual falls only, which yielded broadly consistent results. These findings support the robustness of our main conclusions while justifying the inclusion of near-falls as part of the primary endpoint.

In addition, from a clinical perspective, distinguishing between isolated and recurrent falls is clinically important because these patterns may reflect different levels of physiological vulnerability (65). Recurrent falls often indicate more severe and progressive deterioration in functional reserve, along with persistent impairment in postural control. Compared with isolated fall events, recurrent falls are associated with markedly increased risks of fragility fractures, hospitalization, and mortality in the older population (66, 67). Although the present study employed a binary outcome framework that does not capture the frequency or temporal dynamics of falls, this approach was deliberately adopted to maximize its clinical utility in routine dialysis care. The primary objective was to develop a tool for early risk identification rather than a longitudinal prognostic model. Accordingly, the proposed CAT-based model is best regarded as a first-line triage tool for identifying patients who may benefit from timely preventive interventions, such as medication review and environmental safety interventions, during routine dialysis sessions. Therefore, the model should be interpreted as an initial risk-stratification tool for screening the broader MHD population and identifying individuals at high risk who may subsequently require comprehensive geriatric assessment or targeted gait and balance interventions. By focusing on short-term (6-month) risk prediction, the model may offer an actionable window for intervention before transient instability progresses to recurrent falls.

### Clinical implications

Beyond statistical performance, the practical value of this model lies in its potential to support routine short-term fall-risk stratification in older adults receiving MHD. Because the final predictors are derived from routinely collected clinical and functional parameters, the model could be incorporated into periodic dialysis reviews or standard geriatric assessments without requiring specialized testing. Importantly, the nine predictive factors in the final model—such as age, using of walking aids, frailty status, comorbid conditions, and functional or cognitive assessments—are routinely obtained during standard clinical evaluations or dialysis care processes. These variables do not require specialized equipment or advanced testing and are commonly documented in electronic medical records or geriatric assessments. This accessibility enhances the feasibility of implementing the model in diverse clinical settings, including resource-limited environments, and supports its potential for broader clinical adoption. To facilitate individualized bedside risk estimation, we translated the final CAT model into a Shiny-based web calculator, enabling real-time, personalized risk stratification.

Crucially, this model is intended to function as an early-warning decision-support aid rather than a replacement for clinical judgment. The individualized risk output may help guide the prioritization of clinical resources. For example, patients identified as having an elevated predicted risk may benefit from targeted preventive interventions, including frailty and mobility reassessments, medication review, optimization of walking-aid use, environmental safety counseling, closer follow-up, and referral for physical rehabilitation or balance-focused interventions when appropriate. Nevertheless, the clinical implications of these findings should be interpreted cautiously. Although DCA suggested potential net benefit, this remains a statistical estimate rather than direct evidence of improved patient outcomes. Accordingly, the model and accompanying web calculator should be regarded as adjunctive tools. Their real-world effectiveness in reducing fall-related events cannot be established from internal validation alone. Prospective implementation studies, ideally including external validation and pragmatic clinical evaluation, are needed before routine clinical adoption can be recommended.

### Limitations

Several limitations should be acknowledged. First, this was a single-center study, and the proposed model underwent internal rather than external validation. Although internal validation demonstrated stable predictive performance within the source population, dialysis populations and clinical practice patterns may vary across regions. Therefore, the generalizability of our findings remains uncertain. External validation and necessary model recalibration in independent multicenter cohorts are needed before broader clinical application.

Second, the primary outcome was defined as a binary composite endpoint of at least one fall or near-fall during 6 months of follow-up. Although this approach is clinically practical for screening purposes, it does not distinguish between single and recurrent events, nor does it capture the time to first fall or the overall event burden. In addition, the 6-month follow-up period was deliberately selected as a short-term prediction horizon to enhance clinical applicability and better reflect patients’ baseline status in routine clinical care; however, this relatively short timeframe may not fully capture longer-term fall trajectories. Future studies incorporating longer follow-up periods and alternative modeling strategies—such as time-to-event, recurrent-event, or count-based approaches—may provide a more comprehensive characterization of fall patterns and risk progression among older adults undergoing MHD.

Third, near-falls may reflect impaired balance or instability but do not necessarily result in injury or functional consequences comparable to actual falls. Therefore, treating near-falls and falls as an equivalent outcome may have introduced outcome heterogeneity and affected the clinical interpretability of the model. Future studies should consider applying hierarchical or multi-state modeling approaches to better distinguish their respective predictors and clinical implications in older adults undergoing MHD.

Fourth, participants with incomplete follow-up were excluded from the primary binary analysis because 6-month outcome ascertainment was unavailable. Although baseline comparisons revealed no statistically significant differences between included and excluded participants, the possibility of selection bias cannot be entirely ruled out. Specifically, the competing risk of death may introduce survival bias, potentially resulting in an underestimation of fall risks among the frailest patients, who may die before a fall event is recorded. Furthermore, patients lost to follow-up may have experienced acute hospitalization or transitioned to long-term care facilities due to sudden functional decline or undocumented severe falls. Consequently, the final analytical cohort may inadvertently represent a relatively more stable subset of the older MHD population. These potential biases should be considered when interpreting the model’s predicted probabilities, particularly for highly unstable or terminally ill patients.

Finally, although the identified geriatric and functional predictors demonstrated good predictive value, the relative lack of explicitly dialysis-related parameters in the final model may limit the disease-specific granularity of our conclusions. Our dataset primarily captured baseline characteristics rather than highly granular, session-to-session dynamic parameters. Potentially relevant dialysis-induced triggers—such as detailed intradialytic hemodynamic fluctuations, precise fluid-shift metrics, and post-dialysis orthostatic hypotension profiles—were not comprehensively available. Future studies should evaluate whether the incorporation of such high-resolution longitudinal dialysis-specific parameters can further improve the specificity and transportability of fall-prediction models in the MHD population.

## Conclusion

In conclusion, we successfully developed and internally validated an interpretable CAT-based ML model for predicting the 6-month risk of fall-related events in older adults receiving MHD. The model demonstrated favorable overall statistical performance and identified clinically meaningful predictors, particularly frailty, using of walking aids, and advanced age. To facilitate practical application at the point of care, we deployed this model as an accessible Shiny-based web calculator. While these findings suggest this tool has the potential to serve as a valuable decision-support aid for short-term risk stratification, external multicenter validation and prospective implementation studies are still required to confirm its true clinical utility and impact on patient outcomes before routine adoption.

## Data Availability

The raw data supporting the conclusions of this article will be made available by the authors, without undue reservation.

## References

[1] Li Z He R Wang Y Qu Z Liu J Yu R . Global trends of chronic kidney disease from 1990 to 2021: a systematic analysis for the global burden of disease study 2021. BMC Nephrol. (2025) 26:385. doi: 10.1186/s12882-025-04309-7, 40660180 PMC12257723

[2] Deng L Guo S Liu Y Zhou Y Liu Y Zheng X . Global, regional, and national burden of chronic kidney disease and its underlying etiologies from 1990 to 2021: a systematic analysis for the global burden of disease study 2021. BMC Public Health. (2025) 25:636. doi: 10.1186/s12889-025-21851-z, 39962443 PMC11831764

[3] Ding X. Chronic kidney disease in China: addressing the burden with innovative strategies and global insights. Diabetes Metab Res Rev. (2025) 41:e70092. doi: 10.1002/dmrr.70092, 41115151

[4] Romagnani P Agarwal R Chan JCN Levin A Kalyesubula R Karam S . Chronic kidney disease. Nat Rev Dis Primers. (2025) 11:8. doi: 10.1038/s41572-024-00589-9, 39885176

[5] Chen Z Zhu L Ma C Li J Liu L Xiao W . Frailty risk prediction models in maintenance hemodialysis patients: a systematic review and meta-analysis of model performance and methodological quality. Ren Fail. (2025) 47:2522329. doi: 10.1080/0886022X.2025.2522329, 40624454 PMC12239115

[6] Arhuidese IJ Beaulieu RJ Aridi HD Locham S Baldwin EK Malas MB. Age-related outcomes of arteriovenous grafts for hemodialysis access. J Vasc Surg. (2020) 72:643–50. doi: 10.1016/j.jvs.2019.10.096, 32067881

[7] Smit F van Zwieten A Sherrington C Franco MR Cullati S Blyth FM . Socioeconomic position across the life course and falls among middle- and older-aged adults: protocol for a systematic review. BMJ Open. (2025) 15:e087971. doi: 10.1136/bmjopen-2024-087971, 39842932 PMC11784422

[8] Salari N Darvishi N Ahmadipanah M Shohaimi S Mohammadi M. Global prevalence of falls in the older adults: a comprehensive systematic review and meta-analysis. J Orthop Surg Res. (2022) 17:334. doi: 10.1186/s13018-022-03222-1, 35765037 PMC9238111

[9] Carvalho TC Dini AP. Risk of falls in people with chronic kidney disease and related factors. Rev Lat Am Enfermagem. (2020) 28:e3289. doi: 10.1590/1518-8345.3911.3289, 32520242 PMC7282714

[10] Tang J Wang B Yuan Q Li X. Prevalence and risk factors of falls in people on hemodialysis: a systematic review and meta-analysis. Ren Fail. (2025) 47:2485375. doi: 10.1080/0886022X.2025.2485375, 40204427 PMC11983538

[11] Hall RK Landerman LR O' Hare AM Anderson RA Colón-Emeric CS. Chronic kidney disease and recurrent falls in nursing home residents: a retrospective cohort study. Geriatr Nurs. (2015) 36:136–41. doi: 10.1016/j.gerinurse.2014.12.012, 25616732 PMC4393772

[12] Goto NA Weststrate ACG Oosterlaan FM Verhaar MC Willems HC Emmelot-Vonk MH . The association between chronic kidney disease, falls, and fractures: a systematic review and meta-analysis. Osteoporos Int. (2020) 31:13–29. doi: 10.1007/s00198-019-05190-5, 31720721 PMC6946749

[13] Shirai N Usui N Okamura D Sato Y Kojima S Mikami K . Relationship between frailty, as assessed using the Kihon checklist, and falls in Hemodialysis patients: a Multicenter prospective cohort study. J Ren Nutr. (2025) 35:655–62. doi: 10.1053/j.jrn.2025.03.006, 40157655

[14] Cui Y Zhou J Liu Q Ye H Liu B. The mediating role of intrinsic capacity in balance and falls among older adults. Sci Rep. (2025) 15:11732. doi: 10.1038/s41598-025-96081-9, 40188249 PMC11972392

[15] Kim EA Mordiffi SZ Bee WH Devi K Evans D. Evaluation of three fall-risk assessment tools in an acute care setting. J Adv Nurs. (2007) 60:427–35. doi: 10.1111/j.1365-2648.2007.04419.x, 17919164

[16] Kehinde JO. Instruments for measuring fall risk in older adults living in long-term care facilities: an integrative review. J Gerontol Nurs. (2009) 35:46–55. doi: 10.3928/00989134-20090902-01, 19772229

[17] Liang XZ Chai JL Li GZ Li W Zhang BC Zhou ZQ . A fall risk prediction model based on the CHARLS database for older individuals in China. BMC Geriatr. (2025) 25:170. doi: 10.1186/s12877-025-05814-y, 40082807 PMC11907985

[18] Liu X Chen S Liu C Dang X Wei M Xin X . Novel risk-factor analysis and risk-evaluation model of falls in patients receiving maintenance hemodialysis. Ren Fail. (2023) 45:2182608. doi: 10.1080/0886022X.2023.2182608, 36856312 PMC9980417

[19] Li L Xu W Fang Y Jiang Q Zhou Y Chen Y . Construction and validation of a fall risk prediction model in elderly maintenance hemodialysis patients: a multicenter prospective cohort study. Ren Fail. (2025) 47:2455524. doi: 10.1080/0886022X.2025.2455524, 39962636 PMC11837925

[20] Breiman L. Statistical Modeling: the two cultures (with comments and a rejoinder by the author). Stat Sci. (2001) 16:199–231. doi: 10.1214/ss/1009213726

[21] Bzdok D Altman N Krzywinski M. Statistics versus machine learning. Nat Methods. (2018) 15:233–4. doi: 10.1038/nmeth.4642, 30100822 PMC6082636

[22] Edwards AS Kaplan B Jie T. A primer on machine learning. Transplantation. (2021) 105:699–703. doi: 10.1097/TP.0000000000003316, 32826799

[23] Usmani S Saboor A Haris M Khan MA Park H. Latest research trends in fall detection and prevention using machine learning: a systematic review. Sensors (Basel). (2021) 21:5134. doi: 10.3390/s21155134, 34372371 PMC8347190

[24] Sharma V Kulkarni V Joon T Eurich DT Simpson SH Voaklander D . Predicting falls-related admissions in older adults in Alberta, Canada: a machine-learning falls prevention tool developed using population administrative health data. BMJ Open. (2023) 13:e071321. doi: 10.1136/bmjopen-2022-071321, 37607796 PMC10445355

[25] Hsu W Elmore JG. Shining light into the black box of machine learning. J Natl Cancer Inst. (2019) 111:877–9. doi: 10.1093/jnci/djy226, 30629201 PMC6748732

[26] Borson S Scanlan J Brush M Vitaliano P Dokmak A. The mini-cog: a cognitive 'vital signs' measure for dementia screening in multi-lingual elderly. Int J Geriatr Psychiatry. (2000) 15:1021–7. doi: 10.1002/1099-1166(200011)15:11<1021::aid-gps234>3.0.co;2-6, 11113982

[27] Dong P Cheng C Yin W Li Z Shi Y Gao M . Frailty as a mediator between sleep quality and cognitive impairment among the rural older adults: a cross-sectional study. BMC Geriatr. (2025) 25:7. doi: 10.1186/s12877-024-05657-z, 39754045 PMC11697922

[28] Buysse DJ Reynolds CF I Monk TH Berman SR Kupfer DJ. The Pittsburgh sleep quality index: a new instrument for psychiatric practice and research. Psychiatry Res. (1989) 28:193–213. doi: 10.1016/0165-1781(89)90047-4 2748771

[29] Wang W Bian Q Zhao Y Li X Wang W Du J . Reliability and validity of the Chinese version of the patient health questionnaire (PHQ-9) in the general population. Gen Hosp Psychiatry. (2014) 36:539–44. doi: 10.1016/j.genhosppsych.2014.05.021 25023953

[30] Kroenke K Spitzer RL Williams JB. The PHQ-9: validity of a brief depression severity measure. J Gen Intern Med. (2001) 16:606–13. doi: 10.1046/j.1525-1497.2001.016009606.x, 11556941 PMC1495268

[31] Spitzer RL Kroenke K Williams JB Löwe B. A brief measure for assessing generalized anxiety disorder: the GAD-7. Arch Intern Med. (2006) 166:1092–7. doi: 10.1001/archinte.166.10.1092, 16717171

[32] Leung SO Chan CC Shah S. Development of a Chinese version of the modified Barthel index-- validity and reliability. Clin Rehabil. (2007) 21:912–22. doi: 10.1177/0269215507077286, 17981850

[33] Lamb SE Jørstad-Stein EC Hauer K Becker C. Development of a common outcome data set for fall injury prevention trials: the prevention of falls network Europe consensus. J Am Geriatr Soc. (2005) 53:1618–22. doi: 10.1111/j.1532-5415.2005.53455.x, 16137297

[34] Maidan I Freedman T Tzemah R Giladi N Mirelman A Hausdorff JM. Introducing a new definition of a near fall: intra-rater and inter-rater reliability. Gait Posture. (2014) 39:645–7. doi: 10.1016/j.gaitpost.2013.07.123, 23972512 PMC3842362

[35] Wu Y Jiang X Wang D Xu L Sun H Xie B . Dynamic nomogram for predicting the fall risk of stroke patients: An observational study. Clin Interv Aging. (2025) 20:197–212. doi: 10.2147/CIA.S486252, 40028259 PMC11871932

[36] Mera-Gaona M Neumann U Vargas-Canas R López DM. Evaluating the impact of multivariate imputation by MICE in feature selection. PLoS One. (2021) 16:e0254720. doi: 10.1371/journal.pone.0254720, 34320016 PMC8318311

[37] Takefuji Y. Mitigating biases in feature selection and importance assessments in predictive models using LASSO regression. Oral Oncol. (2024) 159:107090. doi: 10.1016/j.oraloncology.2024.107090, 39488166

[38] An S Ye Z Che W Gao Y Li J Zheng J. Development and validation of machine learning models to predict in-hospital mortality in ICU patients with sepsis and chronic kidney disease. BMC Infect Dis. (2025) 25:1504. doi: 10.1186/s12879-025-11949-5, 41194045 PMC12587723

[39] Lundberg SM Erion G Chen H DeGrave A Prutkin JM Nair B . From local explanations to global understanding with explainable AI for trees. Nat Mach Intell. (2020) 2:56–67. doi: 10.1038/s42256-019-0138-9, 32607472 PMC7326367

[40] Abdel-Rahman EM Yan G Turgut F Balogun RA. Long-term morbidity and mortality related to falls in hemodialysis patients: role of age and gender - a pilot study. Nephron Clin Pract. (2011) 118:c278–84. doi: 10.1159/000322275, 21212691

[41] Stoltzfus JC. Logistic regression: a brief primer. Acad Emerg Med. (2011) 18:1099–104. doi: 10.1111/j.1553-2712.2011.01185.x, 21996075

[42] Uddin S Khan A Hossain ME Moni MA. Comparing different supervised machine learning algorithms for disease prediction. BMC Med Inform Decis Mak. (2019) 19:281. doi: 10.1186/s12911-019-1004-8, 31864346 PMC6925840

[43] Polus JS Bloomfield RA Vasarhelyi EM Lanting BA Teeter MG. Machine learning predicts the fall risk of Total hip arthroplasty patients based on wearable sensor instrumented performance tests. J Arthroplast. (2021) 36:573–8. doi: 10.1016/j.arth.2020.08.034, 32928593

[44] Hand DJ Yu K. Idiot's bayes—not so stupid after all? Int Stat Rev. (2001) 69:385–98. doi: 10.1111/j.1751-5823.2001.tb00465.x, 40046247

[45] Jabeur SB Gharib C Mefteh-Wali S Arfi WB. CatBoost model and artificial intelligence techniques for corporate failure prediction. Technol Forecast Soc Change. (2021) 166:120658. doi: 10.1016/j.techfore.2021.120658

[46] Zhang C Liu L. Machine learning prediction model for medical environment comfort based on SHAP and LIME interpretability analysis. Sci Rep. (2025) 15:39269. doi: 10.1038/s41598-025-22972-6, 41214038 PMC12603039

[47] McAdams-DeMarco MA Tan J Salter ML Gross A Meoni LA Jaar BG . Frailty and cognitive function in incident hemodialysis patients. Clin J Am Soc Nephrol. (2015) 10:2181–9. doi: 10.2215/CJN.01960215, 26573615 PMC4670760

[48] Abdel-Rahman EM Turgut F Turkmen K Balogun RA. Falls in elderly hemodialysis patients. QJM. (2011) 104:829–38. doi: 10.1093/qjmed/hcr108, 21750022

[49] Cook WL Tomlinson G Donaldson M Markowitz SN Naglie G Sobolev B . Falls and fall-related injuries in older dialysis patients. Clin J Am Soc Nephrol. (2006) 1:1197–204. doi: 10.2215/CJN.01650506, 17699348

[50] Tsai YJ Yang PY Yang YC Lin MR Wang YW. Prevalence and risk factors of falls among community-dwelling older people: results from three consecutive waves of the national health interview survey in Taiwan. BMC Geriatr. (2020) 20:529. doi: 10.1186/s12877-020-01922-z, 33297968 PMC7724833

[51] Chen X He L Shi K Yang J Du X Shi K . Age-stratified modifiable fall risk factors in Chinese community-dwelling older adults. Arch Gerontol Geriatr. (2023) 108:104922. doi: 10.1016/j.archger.2023.104922, 36634440

[52] Roman de Mettelinge T Cambier D. Understanding the relationship between walking aids and falls in older adults: a prospective cohort study. J Geriatr Phys Ther. (2015) 38:127–32. doi: 10.1519/JPT.0000000000000031, 25594520

[53] Ishii T Matsumoto W Hoshino Y Kagawa Y Iwasaki E Takada H . Walking aids and complicated orthopedic diseases are risk factors for falls in hemodialysis patients: an observational study. BMC Geriatr. (2023) 23:319. doi: 10.1186/s12877-023-04015-9, 37217875 PMC10204155

[54] Zak M Sikorski T Wasik M Courteix D Dutheil F Brola W. Frailty syndrome-fall risk and rehabilitation management aided by virtual reality (VR) technology solutions: a narrative review of the current literature. Int J Environ Res Public Health. (2022) 19:2985. doi: 10.3390/ijerph19052985, 35270677 PMC8910391

[55] Delgado C Shieh S Grimes B Chertow GM Dalrymple LS Kaysen GA . Association of Self-Reported Frailty with falls and fractures among patients new to Dialysis. Am J Nephrol. (2015) 42:134–40. doi: 10.1159/000439000, 26381744 PMC4596065

[56] Gabay C Ruedin P Slosman D Bonjour JP Leski M Rizzoli R. Bone mineral density in patients with end-stage renal failure. Am J Nephrol. (1993) 13:115–23. doi: 10.1159/000168600, 8342576

[57] Chiu E Markowitz SN Cook WL Jassal SV. Visual impairment in elderly patients receiving long-term hemodialysis. Am J Kidney Dis. (2008) 52:1131–8. doi: 10.1053/j.ajkd.2008.05.032, 18706752

[58] Wang HH Wu JL Lee YC Ho LC Chang MY Liou HH . Risk of serious falls between Hemodialysis and peritoneal Dialysis patients: a Nationwide population-based cohort study. Sci Rep. (2020) 10:7799. doi: 10.1038/s41598-020-64698-7, 32385311 PMC7211016

[59] Vande Walle N Kenis C Heeren P Van Puyvelde K Decoster L Beyer I . Fall predictors in older cancer patients: a multicenter prospective study. BMC Geriatr. (2014) 14:135. doi: 10.1186/1471-2318-14-135, 25511244 PMC4320446

[60] Wei X Peng J Chang R Liu Q. Prevalence and influencing factors of cognitive frailty in Chinese maintenance hemodialysis patients: a systematic review and meta-analysis. BMC Nephrol. (2025) 26:365. doi: 10.1186/s12882-025-04288-9, 40629315 PMC12235981

[61] McIntyre CW Jain A. Dialysis and cognitive impairment. Nat Rev Nephrol. (2025) 21:553–64. doi: 10.1038/s41581-025-00960-3, 40275017

[62] Tavares GMS Pacheco BP Gottlieb MGV Müller DVK Santos GM. Interaction between cognitive status, fear of falling, and balance in elderly persons. Clinics (Sao Paulo). (2020) 75:e1612. doi: 10.6061/clinics/2020/e1612, 33146348 PMC7561071

[63] Oosting IJ Colombijn JMT Kaasenbrood L Liabeuf S Laville SM Hooft L . Polypharmacy in patients with CKD: a systematic review and Meta-analysis. Kidney360. (2024) 5:841–50. doi: 10.34067/KID.0000000000000447, 38661553 PMC11219116

[64] Nagai K Yamada M Komatsu M Tamaki A Kanai M Miyamoto T . Near falls predict substantial falls in older adults: a prospective cohort study. Geriatr Gerontol Int. (2017) 17:1477–80. doi: 10.1111/ggi.12898, 27577092

[65] Alves EBG Lucchetti ALG Barros AAA de Carvalho Souza SQ Rocha RPR Almeida SM . A comprehensive investigation to examine the factors associated with previous falls, recurrent falls, and concerns about falling among outpatient older individuals: a cross-sectional study. Eur Geriatr Med. (2025) 16:949–62. doi: 10.1007/s41999-025-01199-8, 40257745

[66] Pérez-Ros P Martínez-Arnau FM Orti-Lucas RM Tarazona-Santabalbina FJ. A predictive model of isolated and recurrent falls in functionally independent community-dwelling older adults. Braz J Phys Ther. (2019) 23:19–26. doi: 10.1016/j.bjpt.2018.05.005, 29914855 PMC6546906

[67] Abreu DR Azevedo RC Silva AM Reiners AA Abreu HC. Factors associated with recurrent falls in a cohort of older adults. Ciênc Saúde Colet. (2016) 21:3439–46. doi: 10.1590/1413-812320152111.21512015, 27828577

