# Supplementary material for: Interpretable machine learning-based predictive model for fall risk in older adults receiving maintenance hemodialysis
Source: Front Med (Lausanne). 2026 May 21;13:1802938. doi: 10.3389/fmed.2026.1802938 (PMC13233273; doi:10.3389/fmed.2026.1802938)
Supplement: Supplementary file 1 [file Data_Sheet_1.docx]

**Supplementary Table S1**. Hyperparameters for the nine machine learning algorithms.

| Algorithms | Parameter Name | Hyperparameter Search Range |
| --- | --- | --- |
| Logistical Regression (LR) | alpha (Regularization Type) | 0.4, 0.6, 0.8 |
|  | lambda (Penalty Strength) | 0.1, 0.5, 0.7 |
|  | maxit (Maximum Iterations) | Fixed at 2000 |
| Random Forest (RF) | mtry (Number of Features per Tree) | 3, 5, 7 |
|  | ntree (Number of Trees) | Fixed at 500 |
|  | nodesize (Minimum Samples per Leaf Node) | 1, 5 |
|  | maxnodes (Maximum Number of Leaf Nodes) | 100, 200 |
|  | sampsize (Sample Size per Tree) | 500, 600 |
| Extreme Gradient Boosting (XGBoost) | max_depth (Tree Depth) | Fixed at 2 |
|  | eta (Learning Rate) | Fixed at 0.03 |
|  | subsample (Sample Sampling Rate) | 0.4, 0.6 |
|  | colsample_bytree (Feature Sampling Rate per Tree) | 0.4, 0.6 |
|  | reg_alpha | Fixed at 1.0 |
|  | reg_lambda | 5.0, 10.0 |
|  | gamma | 1.0, 2.0 |
|  | scale_pos_weight | Fixed at 0.9 |
|  | nrounds | Fixed at 200 |
| Light Gradient Boosting Machine (LGBM) | num_leaves (Number of Leaf Nodes) | 2, 4 |
|  | learning_rate | Fixed at 0.05 |
|  | reg_lambda | 1.0, 2.0, 3 |
|  | subsample | 0.3, 0.5 |
|  | colsample_bytree | Fixed at 0.7 |
|  | min_data_in_leaf (Minimum Data in Leaf Node) | Fixed at 40 |
|  | min_sum_hessian_in_leaf | Fixed at 35 |
|  | reg_alpha | Fixed at 0.3 |
| Naïve Bayes (NB) | fL (Laplacian Smoothing) | 1, 2, 3 |
|  | usekernel (Kernel Function Switch) | TRUE/FALSE |
|  | adjust (Kernel Function Adjustment Coefficient) | 0.8, 1.0, 1.2 |
| Categorical Boosting (CAT) | depth (Tree Depth) | 2, 3 |
|  | iterations | Fixed at 300 |
|  | learning_rate | Fixed at 0.03 |
|  | class_weights_neg (Negative Class Weight) | 1.1, 1.3 |
|  | reg_lambda | 9, 11 |
|  | mfinal (Number of Iterations/Trees) | 10, 20, 30, 50 |
|  | maxdepth (Base Tree Depth) | 2, 4, 6 |
| Support Vector Machine (SVM) | C (Penalty Coefficient) | 1, 2, 3 |
|  | sigma (RBF Kernel Bandwidth) | 0.03, 0.05, 0.1 |
| Extra Trees (ET) | mtry (Number of Features per Tree) | 2, 3 |
|  | min.node.size (Minimum Samples per Leaf Node) | 15, 25 |
|  | num.trees (Number of Trees) | Fixed at 500 |
|  | sample.fraction (Sampling Fraction) | Fixed at 0.55 |

**Supplementary Table S2** Comparison between included and death or loss follow up patients.

| Characteristics | Included  (N = 1,248) | Excluded  (N = 133) | Statistics | *p*-value |
| --- | --- | --- | --- | --- |
| Age | 73.00 [69.00, 78.00] | 75.00 [69.50, 79.00] | -1.933 | 0.053 |
| Gender |  |  |  |  |
| Male | 758 (60.7%) | 84 (63.2%) | 0.296 | 0.586 |
| Female | 490 (39.3%) | 49 (36.8%) |  |  |
| BMI (kg/m^2^) | 23.30 [20.00, 27.90] | 23.30 [20.40, 27.70] | -0.189 | 0.850 |
| Education level |  |  |  |  |
| Below high school | 910 (72.9%) | 94 (70.7%) | 0.304 | 0.581 |
| High school or above | 338 (27.1%) | 39 (29.3%) |  |  |
| Marital status |  |  |  |  |
| Married | 835 (66.9%) | 88 (66.2%) | 0.030 | 0.863 |
| Single/divorced/widowed | 413 (33.1%) | 45 (33.8%) |  |  |
| Caregivers |  |  |  |  |
| Family member | 1,129 (90.5%) | 120 (90.2%) | 0.008 | 0.929 |
| Nursing assistant or others | 119 (9.5%) | 13 (9.8%) |  |  |
| Monthly income |  |  |  |  |
| <3000 RMB | 552 (44.2%) | 62 (46.6%) |  |  |
| 3000-5000 RMB | 568 (45.5%) | 58 (43.6%) | 0.277 | 0.871 |
| >5000 RMB | 128 (10.3%) | 13 (9.8%) |  |  |
| Current smoking | 116 (9.3%) | 15 (11.3%) | 0.551 | 0.458 |
| Current alcohol consumption | 95 (7.6%) | 12 (9.0%) | 0.334 | 0.563 |
| Dialysis duration |  |  |  |  |
| <2 years | 272 (21.8%) | 31 (23.3%) | 0.161 | 0.923 |
| 2-5 years | 593 (47.5%) | 62 (46.6%) |  |  |
| >5 years | 383 (30.7%) | 40 (30.1%) |  |  |
| Dialysis frequency per week |  |  |  |  |
| ≤2 times | 325 (26.0%) | 39 (29.3%) | 0.667 | 0.414 |
| >2 times | 923 (74.0%) | 94 (70.7%) |  |  |
| Vascular access type |  |  |  |  |
| Arteriovenous fistula | 901 (72.2%) | 87 (65.4%) | 2.715 | 0.099 |
| Central venous catheter | 347 (27.8%) | 46 (34.6%) |  |  |
| Intradialytic hypotension | 301 (24.1%) | 36 (27.1%) | 0.567 | 0.452 |
| Diabetes | 478 (38.3%) | 57 (42.9%) | 1.051 | 0.305 |
| Hypertension | 986 (79.0%) | 100 (75.2%) | 1.043 | 0.307 |
| Stroke | 215 (17.2%) | 26 (19.5%) | 0.450 | 0.503 |
| Cardiovascular disease | 325 (26.0%) | 35 (26.3%) | 0.005 | 0.945 |
| Orthopedic disease | 551 (44.2%) | 60 (45.1%) | 0.045 | 0.832 |
| Sedative-hypnotic medications | 439 (35.2%) | 45 (33.8%) | 0.095 | 0.758 |
| Polypharmacy | 1,106 (88.6%) | 112 (84.2%) | 2.247 | 0.134 |
| Using of walking aids | 461 (36.9%) | 51 (38.3%) | 0.102 | 0.749 |
| History of falls in the past year | 352 (28.2%) | 39 (29.3%) | 0.074 | 0.786 |
| Visual impairment | 716 (57.4%) | 75 (56.4%) | 0.047 | 0.828 |
| Hearing impairment | 411 (32.9%) | 47 (35.3%) | 0.314 | 0.575 |
| Cognitive impairment | 353 (28.3%) | 35 (26.3%) | 0.231 | 0.631 |
| Poor sleep quality | 947 (75.9%) | 102 (76.7%) | 0.043 | 0.835 |
| Frailty | 521 (41.7%) | 63 (47.4%) | 1.556 | 0.212 |
| Depression | 778 (62.3%) | 82 (61.7%) | 0.024 | 0.877 |
| Anxiety | 721 (57.8%) | 75 (56.4%) | 0.094 | 0.759 |
| ADL dependency | 835 (66.9%) | 93 (69.9%) | 0.497 | 0.481 |
| Laboratory parameters |  |  |  |  |
| Hemoglobin (g/L) | 114.50 [100.00, 132.00] | 113.00 [98.00, 129.00] | -0.881 | 0.378 |
| Albumin (g/L) | 38.00 [31.00, 44.00] | 38.00 [32.00, 44.00] | -0.100 | 0.920 |
| Serum potassium (mmol/L) | 4.70 [4.20, 5.20] | 4.80 [4.00, 5.20] | -0.052 | 0.958 |
| Serum phosphate (mmol/L) | 1.61 [1.38, 1.98] | 1.59 [1.39, 2.02] | -0.075 | 0.941 |
| Serum calcium (mmol/L) | 1.78 [1.57, 2.00] | 1.75 [1.58, 2.00] | -0.304 | 0.761 |
| Serum sodium (mmol/L) | 142.00 [138.00, 146.00] | 142.00 [139.00, 146.00] | -0.794 | 0.427 |
| iPTH (pg/mL) | 249.00 [162.00, 377.75] | 236.00 [168.00, 331.00] | -1.205 | 0.228 |
| CRP (mg/L) | 8.52 [6.17, 10.52] | 8.81 [7.07, 10.88] | -1.077 | 0.281 |
| TC (mmol/L) | 4.10 [3.50, 4.80] | 4.10 [3.60, 4.60] | -0.317 | 0.751 |
| TG (mmol/L) | 1.80 [1.60, 2.10] | 1.80 [1.70, 2.10] | -0.677 | 0.498 |
| BUN (mmol/L) | 21.70 [17.70, 27.10] | 21.60 [18.70, 25.80] | -0.219 | 0.827 |
| UA (mg/dL) | 9.60 [8.10, 11.10] | 9.80 [8.20, 11.10] | -0.441 | 0.659 |
| Scr (mg/dL) | 7.50 [5.80, 9.20] | 7.50 [6.20, 9.40] | -1.017 | 0.309 |

**Notes.** Data are presented as medians [interquartile ranges] or N (%).

BMI: Body mass index; RMB: Renminbi; ADL: Activities of daily living; iPTH: Intact parathyroid hormone; CRP: C-reactive protein; TC: Total cholesterol; TG: Triglyceride; BUN: Blood urea nitrogen; UA: Uric acid; Scr: Serum creatinine.

**Supplementary Table S3** Comparison of baseline characteristics between patients in the training and testing sets.

| Characteristics | Total  (N = 1,248) | Training set (N = 874) | Testing set (N = 374) | Statistics | *p*-value |
| --- | --- | --- | --- | --- | --- |
| Age | 73.00 [69.00, 78.00] | 73.00 [69.00, 78.00] | 73.00 [69.00, 79.00] | -0.259 | 0.796 |
| Gender |  |  |  |  |  |
| Male | 758 (60.7%) | 529 (60.5%) | 229 (61.2%) | 0.054 | 0.816 |
| Female | 490 (39.3%) | 345 (39.5%) | 145 (38.8%) |  |  |
| BMI (kg/m^2^) | 23.30 [20.00, 27.90] | 23.35 [20.20, 28.10] | 23.05 [19.78, 27.70] | -1.158 | 0.247 |
| Education level |  |  |  |  |  |
| Below high school | 910 (72.9%) | 647 (74.0%) | 263 (70.3%) | 1.822 | 0.177 |
| High school or above | 338 (27.1%) | 227 (26.0%) | 111 (29.7%) |  |  |
| Marital status |  |  |  |  |  |
| Married | 835 (66.9%) | 590 (67.5%) | 245 (65.5%) | 0.472 | 0.492 |
| Single/divorced/widowed | 413 (33.1%) | 284 (32.5%) | 129 (34.5%) |  |  |
| Caregivers |  |  |  |  |  |
| Family member | 1,129 (90.5%) | 796 (91.1%) | 333 (89.0%) | 1.261 | 0.261 |
| Nursing assistant or others | 119 (9.5%) | 78 (8.9%) | 41 (11.0%) |  |  |
| Monthly income |  |  |  |  |  |
| <3000 RMB | 552 (44.2%) | 380 (43.5%) | 172 (46.0%) | 1.879 | 0.391 |
| 3000-5000 RMB | 568 (45.5%) | 398 (45.5%) | 170 (45.5%) |  |  |
| >5000 RMB | 128 (10.3%) | 96 (11.0%) | 32 (8.6%) |  |  |
| Current smoking | 116 (9.3%) | 81 (9.3%) | 35 (9.4%) | 0.003 | 0.960 |
| Current alcohol consumption | 95 (7.6%) | 67 (7.7%) | 28 (7.5%) | 0.012 | 0.913 |
| Dialysis duration |  |  |  |  |  |
| <2 years | 272 (21.8%) | 183 (20.9%) | 89 (23.8%) | 3.661 | 0.160 |
| 2-5 years | 593 (47.5%) | 409 (46.8%) | 184 (49.2%) |  |  |
| >5 years | 383 (30.7%) | 282 (32.3%) | 101 (27.0%) |  |  |
| Dialysis frequency per week |  |  |  |  |  |
| ≤2 times | 325 (26.0%) | 226 (25.9%) | 99 (26.5%) | 0.051 | 0.821 |
| >2 times | 923 (74.0%) | 648 (74.1%) | 275 (73.5%) |  |  |
| Vascular access type |  |  |  |  |  |
| Arteriovenous fistula | 901 (72.2%) | 626 (71.6%) | 275 (73.5%) | 0.473 | 0.491 |
| Central venous catheter | 347 (27.8%) | 248 (28.4%) | 99 (26.5%) |  |  |
| Intradialytic hypotension | 301 (24.1%) | 211 (24.1%) | 90 (24.1%) | 0.001 | 0.977 |
| Diabetes | 478 (38.3%) | 325 (37.2%) | 153 (40.9%) | 1.537 | 0.215 |
| Hypertension | 986 (79.0%) | 695 (79.5%) | 291 (77.8%) | 0.463 | 0.496 |
| Stroke | 215 (17.2%) | 153 (17.5%) | 62 (16.6%) | 0.158 | 0.691 |
| Cardiovascular disease | 325 (26.0%) | 229 (26.2%) | 96 (25.7%) | 0.039 | 0.844 |
| Orthopedic disease | 551 (44.2%) | 397 (45.4%) | 154 (41.2%) | 1.916 | 0.166 |
| Sedative-hypnotic medications | 439 (35.2%) | 297 (34.0%) | 142 (38.0%) | 1.825 | 0.177 |
| Polypharmacy | 1,106 (88.6%) | 779 (89.1%) | 327 (87.4%) | 0.748 | 0.387 |
| Using of walking aids | 461 (36.9%) | 326 (37.3%) | 135 (36.1%) | 0.163 | 0.687 |
| History of falls in the past year | 352 (28.2%) | 250 (28.6%) | 102 (27.3%) | 0.229 | 0.632 |
| Visual impairment | 716 (57.4%) | 497 (56.9%) | 219 (58.6%) | 0.306 | 0.580 |
| Hearing impairment | 411 (32.9%) | 280 (32.0%) | 131 (35.0%) | 1.060 | 0.303 |
| Cognitive impairment | 353 (28.3%) | 259 (29.6%) | 94 (25.1%) | 2.615 | 0.106 |
| Poor sleep quality | 947 (75.9%) | 674 (77.1%) | 273 (73.0%) | 2.432 | 0.119 |
| Frailty | 521 (41.7%) | 367 (42.0%) | 154 (41.2%) | 0.071 | 0.789 |
| Depression | 778 (62.3%) | 545 (62.4%) | 233 (62.3%) | 0.000 | 0.985 |
| Anxiety | 721 (57.8%) | 517 (59.2%) | 204 (54.5%) | 2.280 | 0.131 |
| ADL dependency | 835 (66.9%) | 591 (67.6%) | 244 (65.2%) | 0.670 | 0.413 |
| Laboratory parameter |  |  |  |  |  |
| Hemoglobin (g/L) | 114.50 [100.00, 132.00] | 114.00 [100.00, 131.00] | 115.50 [100.00, 133.00] | -0.529 | 0.597 |
| Albumin (g/L) | 38.00 [31.00, 44.00] | 38.00 [31.00, 44.00] | 38.00 [32.00, 44.00] | -0.042 | 0.966 |
| Serum potassium (mmol/L) | 4.70 [4.20, 5.20] | 4.70 [4.20, 5.20] | 4.70 [4.20, 5.13] | -0.570 | 0.569 |
| Serum phosphate (mmol/L) | 1.61 [1.38, 1.98] | 1.61 [1.39, 1.96] | 1.61 [1.34, 2.05] | -0.365 | 0.715 |
| Serum calcium (mmol/L) | 1.78 [1.57, 2.00] | 1.79 [1.59, 2.01] | 1.76 [1.54, 1.99] | -1.916 | 0.055 |
| Serum sodium (mmol/L) | 142.00 [138.00, 146.00] | 142.00 [138.00, 146.00] | 142.00 [138.00, 146.00] | -0.157 | 0.875 |
| iPTH (pg/mL) | 249.00 [162.00, 377.75] | 253.50 [165.00, 382.00] | 240.00 [155.00, 371.50] | -1.321 | 0.187 |
| CRP (mg/L) | 8.52 [6.17, 10.52] | 8.46 [6.06, 10.47] | 8.75 [6.70, 10.63] | -1.586 | 0.113 |
| TC (mmol/L) | 4.10 [3.50, 4.80] | 4.10 [3.50, 4.80] | 4.10 [3.50, 4.80] | -0.195 | 0.845 |
| TG (mmol/L) | 1.80 [1.60, 2.10] | 1.80 [1.60, 2.10] | 1.80 [1.60, 2.00] | -0.834 | 0.405 |
| BUN (mmol/L) | 21.70 [17.70, 27.10] | 22.10 [17.80, 27.13] | 20.90 [17.30, 26.95] | -1.102 | 0.271 |
| UA (mg/dL) | 9.60 [8.10, 11.10] | 9.70 [8.18, 11.10] | 9.50 [7.90, 11.00] | -1.503 | 0.133 |
| Scr (mg/dL) | 7.50 [5.80, 9.20] | 7.50 [5.80, 9.20] | 7.45 [5.60, 9.13] | -0.252 | 0.801 |
| Fall events |  |  |  |  |  |
| No | 761 (61.0%) | 530 (60.6%) | 231 (61.8%) | 0.139 | 0.709 |
| Yes | 487 (39.0%) | 344 (39.4%) | 143 (38.2%) |  |  |

**Notes.** Data are presented as medians [interquartile ranges] or N (%).

BMI: Body mass index; RMB: Renminbi; ADL: Activities of daily living; iPTH: Intact parathyroid hormone; CRP: C-reactive protein; TC: Total cholesterol; TG: Triglyceride; BUN: Blood urea nitrogen; UA: Uric acid; Scr: Serum creatinine.

**Supplementary Table S4.** Pre-imputation missing counts for the final selected predictors.

| Variables | Missing number (%) |
| --- | --- |
| Age | / |
| Using of walking aids | / |
| Frailty | 69 (5.5) |
| Orthopedic disease | / |
| Visual impairment | / |
| ADL dependency | / |
| Cognitive impairment | / |
| Stroke | 59 (4.7) |
| Polypharmacy | / |

**Notes.** ADL: Activities of daily living.

**Supplementary Table S5**. Sensitivity analysis of nine ML models for actual falls only in the testing cohort.

| Model | Accuracy | AUC (95% CI) | Recall | Precision | F1 score | Specific-ity | Log  Loss | Brier score |
| --- | --- | --- | --- | --- | --- | --- | --- | --- |
| LR | 0.730 | 0.794 (0.743–0.845) | 0.010 | 1.000 | 0.019 | 1.000 | 0.523 | 0.173 |
| RF | 0.754 | 0.799 (0.750–0.848) | 0.284 | 0.604 | 0.387 | 0.930 | 0.488 | 0.158 |
| XGBoost | 0.749 | 0.812 (0.765–0.859) | 0.137 | 0.700 | 0.230 | 0.978 | 0.484 | 0.160 |
| LGBM | 0.775 | 0.810 (0.763–0.857) | 0.314 | 0.696 | 0.432 | 0.949 | 0.469 | 0.154 |
| NB | 0.727 | 0.814 (0.767–0.861) | 0.029 | 0.500 | 0.056 | 0.989 | 0.598 | 0.195 |
| CAT | 0.751 | 0.816 (0.770–0.862) | 0.176 | 0.667 | 0.279 | 0.967 | 0.472 | 0.158 |
| AdaBoost | 0.773 | 0.804 (0.756–0.853) | 0.294 | 0.698 | 0.414 | 0.952 | 0.494 | 0.161 |
| SVM | 0.757 | 0.810 (0.761–0.860) | 0.225 | 0.657 | 0.336 | 0.956 | 0.496 | 0.162 |
| ET | 0.749 | 0.803 (0.755–0.850) | 0.127 | 0.722 | 0.217 | 0.982 | 0.487 | 0.161 |

Notes. AUC: Area under the receiver operating characteristic curve; CI: Confidence interval; LR: Logistic regression; RF: Random forest; XGBoost: Extreme gradient boosting; LGBM: Light gradient boosting machine; NB: Naive Bayes; CAT: Categorical boosting; AdaBoost: Adaptive boosting; SVM: Support vector machine; ET: Extra trees.

Supplementary Figure S1.


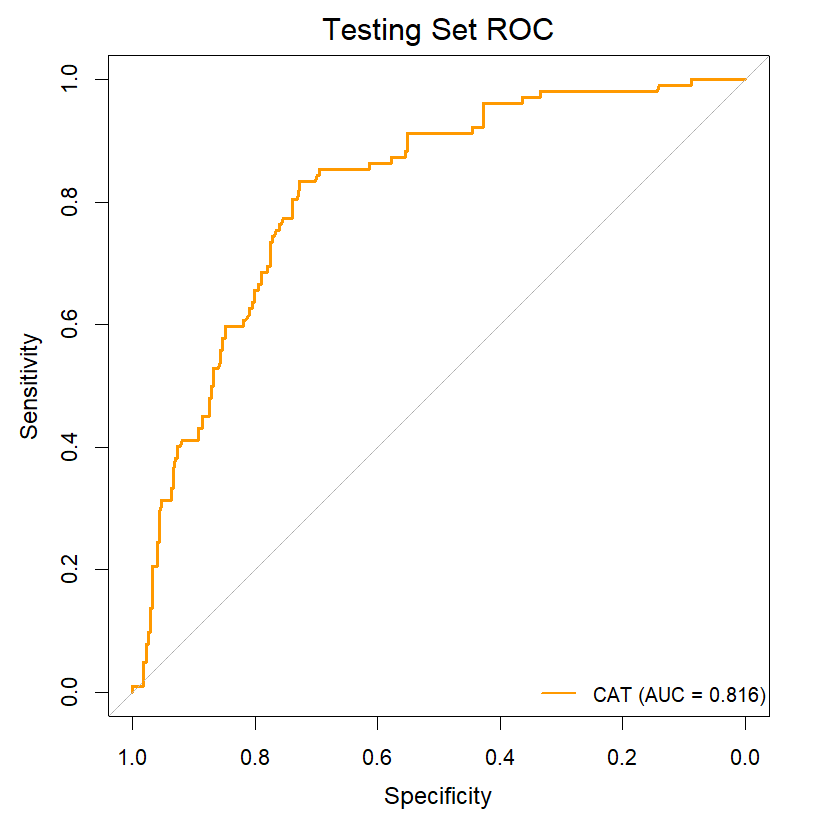


**Supplementary Figure S1.** Sensitivity analysis of ROC in the CAT model for prediction of actual falls only within the independent testing cohort.
